# Sustainable solutions to mitigate occupational heat strain – an umbrella review of physiological effects and global health perspectives

**DOI:** 10.1186/s12940-020-00641-7

**Published:** 2020-09-04

**Authors:** Nathan B. Morris, Ollie Jay, Andreas D. Flouris, Ana Casanueva, Chuansi Gao, Josh Foster, George Havenith, Lars Nybo

**Affiliations:** 1 Department of Nutrition, Exercise and Sports, Section for Integrative Physiology, University of Copenhagen, Copenhagen N, Denmark; 2 Thermal Ergonomics Laboratory, Faculty of Medicine and Health, University of Sydney, Sydney, Australia; 3 FAME Laboratory, School of Exercise Science, University of Thessaly, Thessaly, Greece; 4 Federal Office of Meteorology and Climatology, MeteoSwiss, Zurich Airport, Zurich, Switzerland; 5 Meteorology Group, Department of Applied Mathematics and Computer Sciences, University of Cantabria, Santander, Spain; 6 Thermal Environment Laboratory, Division of Ergonomics and Aerosol Technology, Department of Design Sciences, Faculty of Engineering, Lund University, Lund, Sweden; 7 Environmental Ergonomics Research Centre, Loughborough Design School, Loughborough University, Loughborough, UK

**Keywords:** Occupational medicine, Occupational hygiene, Environmental health, Climate change, Heat stress

## Abstract

**Background:**

Climate change is set to exacerbate occupational heat strain, the combined effect of environmental and internal heat stress on the body, threatening human health and wellbeing. Therefore, identifying effective, affordable, feasible and sustainable solutions to mitigate the negative effects on worker health and productivity, is an increasingly urgent need.

**Objectives:**

To systematically identify and evaluate methods that mitigate occupational heat strain in order to provide scientific-based guidance for practitioners.

**Methods:**

An umbrella review was conducted in biomedical databases employing the following eligibility criteria: 1) ambient temperatures > 28 °C or hypohydrated participants, 2) healthy adults, 3) reported psychophysiological (thermal comfort, heart rate or core temperature) and/or performance (physical or cognitive) outcomes, 4) written in English, and 5) published before November 6, 2019. A second search for original research articles was performed to identify interventions of relevance but lacking systematic reviews. All identified interventions were independently evaluated by all co-authors on four point scales for effectiveness, cost, feasibility and environmental impact.

**Results:**

Following screening, 36 systematic reviews fulfilled the inclusion criteria. The most effective solutions at mitigating occupational heat strain were wearing specialized cooling garments, (physiological) heat acclimation, improving aerobic fitness, cold water immersion, and applying ventilation. Although air-conditioning and cooling garments in ideal settings provide best scores for effectiveness, the limited applicability in certain industrial settings, high economic cost and high environmental impact are drawbacks for these solutions. However, (physiological) acclimatization, planned breaks, shading and optimized clothing properties are attractive alternative solutions when economic and ecological sustainability aspects are included in the overall evaluation.

**Discussion:**

Choosing the most effective solution or combinations of methods to mitigate occupational heat strain will be scenario-specific. However, this paper provides a framework for integrating effectiveness, cost, feasibility (indoors and outdoor) and ecologic sustainability to provide occupational health and safety professionals with evidence-based guidelines.

## Introduction

The increased intensity, frequency and geographical spread of extreme heat events or prolonged periods with high temperatures is a global societal challenge associated with climate change, with widespread health effects, as even moderate elevations of the mean global temperature may cause a disproportional increase in very hot days [1]. Improved heat-warning systems and procedures to prevent deadly exposure of elderly and other vulnerable populations, typically by limiting activity and reducing the temperature of the local indoor environment using air-conditioning, may limit fatalities caused by sudden rises in outdoor temperature [2]. However, for workers, remaining inactive or avoiding heat exposure is incompatible with maintaining productivity, often directly affecting individual income [3]. Further, prolonged periods of elevated temperatures particularly impacts primary (raw materials; e.g. farming and mining) as well as secondary (finished goods; e.g. construction and manufacturing) sector economies [4, 5]; however, smaller reductions in office worker productivity have been reported as well [5]. The resultant socio-economic effects are skewed, as low-income manual workers are most likely to work in physically demanding jobs outdoors [6], thereby specifically affecting occupational health in low-income countries, located in tropical regions, by impeding the ability to prevent poverty and provide affordable health [7]. In addition, those working in the heat have an elevated risk of kidney disorders [8] and increased risk for acute work injuries [9–11], as heat stress may impair cognitive performance [12], elevate the cardiovascular strain for a given activity [13], and accelerate the development of fatigue [12, 14, 15] with all effects further aggravated by dehydration [16].

Human functioning depends on thermal homeostasis and the ability to maintain balance between internal metabolic heat production and heat exchange with the environment. In occupational settings this balance is challenged because the metabolic energy turnover increases when a worker is physically active. Additional industrial heat or protective clothing safety requirements may further aggravate the overall occupational heat strain, comprised of the physiological response to integrated effects of environmental and endogenous heat stress associated with manual labour [17–19]. Endogenous heat production increases in proportion to work intensity; accordingly, occupational heat strain is a pressing issue in industries involving intense activities or occupational settings where workers need to wear protective clothing (e.g. for safety reasons or prevention of exposure to chemical agents), as the high insulation and evaporative resistance of such clothing/safety gear markedly impedes dry and evaporative heat loss [20, 21]. Similarly, superimposed radiative heating, either when working outdoors with exposure to the sun [22] or working around hot machinery [23], can greatly elevate physiological strain and reduce work capacity [24]. Since different combinations of environmental factors, endogenous heat production and insulation and evaporative resistance of clothing can provoke unhealthy and unwarranted occupational heat strain, selecting the appropriate cooling intervention will be scenario specific to best counteract the primary heat stress factors. For example, shading to reduce solar radiation will be very effective for outdoor workers on a sunny day, but not relevant for indoors industries, unless strong radiant heat sources are present. Some interventions may also be feasible in one setting but not applicable in other conditions. For example, skin wetting is effective for/in those with minimal clothing, but not for the worker required to wear encapsulating protective clothing. Furthermore, industries will also consider the economic costs associated with a given solution, to ensure the increases in worker productivity outweigh the implementation costs. Moreover, energy intensive interventions, such as air-conditioning in large production bays, will also be harmful to the environment if the energy sources are non-renewable, thereby worsening the underlying issue of climate change. Therefore, to select the best possible cooling intervention for a given scenario, a wide array of interventions as well as the effectiveness, feasibility, transferability, cost and environmental sustainability must be known in order to provide well-informed advice.

Accordingly, we performed an umbrella review [25] of all systematically analysed interventions and available solutions to mitigate occupational heat strain. To provide a comprehensive identification and evaluation of all potential solutions the meta-analyses review was supplemented by a secondary search to identify additional interventions not yet systematically reviewed. Finally, we evaluated all effective solutions for transferability (applicability and effectiveness in different settings – considering indoor/outdoor industries), how feasibility of implementation in occupational settings, the economic impact (considering acquisition and running cost) and environmental sustainability [26].

## Methods

### Search methods and identification of interventions

In order to identify and evaluate all available interventions investigated for their ability to improve health outcomes or mitigate negative effects of heat stress on physical work capacity or cognitively dominated performances in the heat, two searches of the literature were performed in the following order: 1) a systematic review of systematic reviews and 2) a secondary search of original research (randomized control trials), driven by review references and expert knowledge of the authors. This approach was selected to first compare the highest level of evidence available for each intervention (systematic reviews and meta-analyses), as stipulated by the hierarchy of evidence for health interventions [27] as is presently recommended [25, 28], and as has been performed in other health fields where a sufficient number of systematic reviews have been performed [29–31]. The secondary search was therefore performed in order to include interventions that are known to have been investigated by original research articles but have yet to be systematically compared.

The systematic review of systematic reviews was conducted in PUBMED and Web of Science including articles published by November 6, 2019. The search was conducted using a list of key search terms identified and agreed upon by the authors and organised into a Boolean search strategy (Additional file 1: Appendix 1).

### Eligibility criteria

The eligibility criteria were systematic reviews: 1) which employed randomized or crossover-controlled trials, 2) where ambient temperature was above 28 °C (as cooling interventions become likely beneficial above this temperatures [32]) or where the study participants were hypohydrated (body weight loss of greater than 2%), 3) which studied healthy adults, 4) which reported either a quantitative and/or qualitative synthesis of findings for at least one of the following primary outcomes: physical performance, cognitive performance, thermal comfort, heart rate or core body temperature, 5) published in the English language and 6) published before November 6, 2019. Where systematic reviews included sub-group analyses of different interventional aspects (e.g. internal vs external cooling and pre vs mid cooling) findings from all relevant sub-group analyses were included.

### Selection, data extraction and quality of reviews

The lead author screened retrieved titles and abstracts to identify potentially relevant reviews. Following this process, full texts were assessed independently by the lead author and a second reviewer (either LN, JF or AC) for eligibility. The methodological quality of all reviews was measured using the validated Assessment of Multiple Systematic Reviews (AMSTAR) checklist (Shea et al. 2017). In line with previous systematic reviews of systematic reviews [29–31], studies with a score between 0 and 4 were deemed to be low quality, between 5 and 8 to be of moderate quality, between 9 and 11 to be of high quality. All studies that received a quality score of 5 or higher were subsequently included.

The following data were extracted by the lead author using a predesigned data extraction form: author; year of publication; study aim; inclusion/exclusion criteria; interventions assessed; number of studies/participants; findings relating exercise/work performance, core and skin temperature, heart rate, thermal sensation/comfort and sweat rate. Due to the heterogeneity of the interventions under investigation, no meta-analyses were conducted [30]. However, for reviews in which effect sizes (ES) and confidence intervals were reported, these data were extracted and compiled together into tables and figures displaying the median and range of the effect sizes found.

### Evaluation of interventions for strength of evidence, cost, feasibility, and environmental sustainability

In addition to evaluating the identified interventions based on strength of evidence and effectiveness, all interventions were subsequently evaluated using previously established criteria for evaluating evidence on public health guidelines [26] on four-point scales. The evaluation criteria were strength of evidence, effectiveness, cost, the feasibility in indoor and outdoor/large environments, and the environmental impact.

S*trength of evidence*, as ranked based on the results of the primary and secondary reviews and the hierarchy of research evidence [27]: A score of 0 indicated evidence based upon expert knowledge or non-human based research, 1 indicated evidence based on original research, 2 indicated identified in systematic reviews but not meta-analysed and 3 indicated that the intervention had been identified in a systematic review and had been meta-analysed.

The *effectiveness* score of the intervention, were based upon the Cohen’s d scores from the meta-analyses, when available, or calculated from original research articles and were interpreted using the common values [33], where a score of: 0 was given to interventions which were found to be ineffective or detrimental; 1 was equivalent to a Cohen’s d of 0.2, denoting a small effect; 2 was equivalent to 0.5, denoting a moderate effect; and 3 was equivalent to 0.8 and above, denoting a large effect.

Typically for analyses pertaining to *cost, feasibility, transferability* and *sustainability*, objective data from real world interventions are utilised [34], however, no such studies to date have been conducted for cooling interventions pertaining to occupational heat strain. This information is nevertheless critical for practitioners in order to assess the effectiveness of recommended public/occupational health practices in order to determine which interventions to prioritize [26]. Accordingly, all authors were provided with a spreadsheet with a list of all interventions identified from the literature searches and were asked to independently assign a score for each assessment criteria on a scale from 0 to 3 (see category and criteria descriptions below); or leave the field blank if an informed score could not be provided. Following the return of the assessments, all scores were averaged, and all authors provided a second round with adjustment or confirmation of their agreement on the intervention scores.

The evaluation of the *cost* of the intervention, incorporated both the initial cost required to establish the intervention as well as operational costs. Accordingly, the following four-point numerical scale with descriptive title (in brackets) was used: 0 (none) for no additional cost compared to normal operating procedure to acquire or use the intervention; 1 (low) if there was a small to moderate acquisition cost but no operational costs; 2 (moderate) for small to moderate acquisition and operational costs; and 3 (high) for moderate to large acquisition and operational costs.

The *feasibility* score was based upon whether an intervention could readily be used in occupational settings. The four-point scale consisted of: 0 for interventions that would not disturb the workers normal operating procedures at all (e.g. lowering the air temperature in indoor facilities where industrial heat production is low/not preventing application of AC); − 1 for interventions that would require minor interruptions in the workers regular routine (e.g. having to carry small amounts of additional weight while working or taking small breaks in work); − 2 for interventions which would moderately interrupt the workers (requiring longer breaks [more than 5 min] or interfere with normal work procedures); and − 3 if the intervention would involve a large disruption of work procedures or when a solution was not applicable in the given setting (e.g. interventions that were impossible to implement or would require exceptionally large break times).

The *environmental sustainability* was assessed based on whether the production of the intervention was energy intensive and/or resulted in the production of environmental pollutants, whether the operation of the intervention was energy intensive, as global fuel sources continue to be over 75% non-renewable sources [35], and whether the waste production of the intervention was intensive. Accordingly, a score of 0 (none) was given for no additional environmental impact; 1 (low) for minor impact (e.g. minor waste production or energy use); 2 (moderate) for moderate impact (e.g. moderately resource intensive in production, moderate energy use, production of waste by products); and 3 (high) for large impact (e.g. energy/resource intensive in production, large energy cost for operation, large waste production).

*Transferability* was assessed by evaluating whether the *effectiveness* and *feasibility* were altered depending on whether the intervention was used indoors or outdoors. These fields were evaluated with the same four-point scales used for the *effectiveness* and *feasibility* analyses, with reference to how the intervention would operate in ideal (e.g. using air-conditioning in a small room) compared to least ideal (e.g. using air -conditioning outdoors) conditions.

## Results

**Fig. 1:**
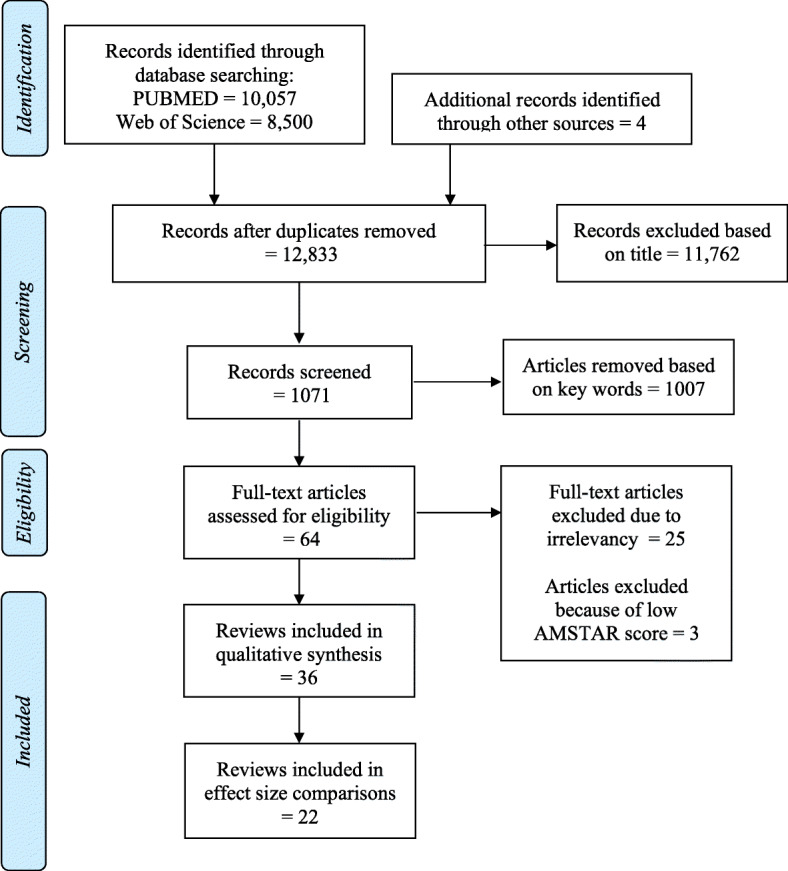
Flow diagram of the review screening process

The systemic search of the databases, detailed in Fig. 1, following the removal of duplicate findings between databases (*n* = 12,829) and the addition of records identified through other sources (*n* = 4) 12,833 unique titles were screened. From these, 11,762 were excluded based on title and 1007 were excluded based on lack of relevant key words (i.e. thermoregulation, temperature, heat). Subsequently, 64 articles were read in full with 10 papers removed as they did not report effects on either health, physiological parameters of importance or productivity effects [36–47]. Furthermore, 13 papers were removed for having some but not all aspects required to be considered a systematic review [48–60] and three articles [61–63] were excluded for having too low quality score (AMSTAR checklist score of 3; see Table 1). As such, 36 articles were included in our final review (Table 2).

**Table 1:** Quality of systematic reviews based on the 11 item AMSTAR checklist

**Article**	Q1	Q2	Q3	Q4	Q5	Q6	Q7	Q8	Q9	Q10	Q11	Total score
**Daanen 2018** [64]	1	1	1	1	1	1	1	1	1	1	1	**11**
**Chalmers 2014** [65]	1	1	1	1	1	1	1	1	1	1	1	**11**
**Jones 2012** [66]	1	1	1	1	1	1	1	1	1	1	1	**11**
**Holland 2017** [67]	1	1	1	1	0	1	1	1	1	1	1	**10**
**McCartney 2017** [68]	1	1	1	1	0	1	1	1	1	1	1	**10**
**Tyler 2016** [69]	1	1	1	1	0	1	1	1	1	1	1	**10**
**Goodman 2019** [70]	1	1	1	1	0	1	1	0	1	1	1	**9**
**Tomes 2017** [71]	1	1	1	1	0	1	1	1	0	1	1	**9**
**Ruddock 2017** [72]	1	0	1	1	0	1	1	1	1	1	1	**9**
**Chan 2015** [73]	1	1	1	1	0	1	1	1	0	1	1	**9**
**Ranalli 2010** [74]	1	1	1	1	1	1	1	1	1	0	0	**9**
**Douzi 2019** [75]	1	0	1	1	0	1	1	1	1	1	0	**8**
**Heffernan 2019** [76]	1	1	1	1	0	0	1	1	0	1	1	**8**
**Jeffries 2019** [77]	1	1	1	1	1	1	0	0	1	0	1	**8**
**Rahimi 2019** [78]	1	1	1	0	0	1	1	1	1	0	1	**8**
**Choo 2018** [79]	1	0	1	1	1	1	1	0	1	0	1	**8**
**Best 2018** [80]	1	1	1	0	0	1	1	1	1	0	1	**8**
**Brearley 2015** [81]	1	0	1	0	1	1	1	1	1	0	1	**8**
**Born 2013** [82]	1	1	1	0	1	1	1	0	1	0	1	**8**
**McEntire 2013** [83]	1	1	1	1	1	1	1	0	1	0	0	**8**
**Burdon 2012** [84]	1	0	1	0	1	1	1	1	1	1	0	**8**
**Goulet 2011** [85]	1	0	1	1	1	1	0	0	1	1	1	**8**
**Vandenbogaerde 2011**	0	0	0	1	1	1	1	1	1	1	1	**8**
**Burdon 2010** [86]	1	1	1	1	1	1	1	0	1	0	0	**8**
**Stearns 2010** [87]	1	1	1	0	1	1	1	1	1	0	0	**8**
**McDermott 2009** [88]	1	1	1	0	1	1	1	1	1	0	0	**8**
**Bongers 2015** [89]	1	1	1	0	0	1	1	0	0	1	1	**7**
**Tyler 2015** [90]	1	0	0	1	1	1	0	0	1	1	1	**7**
**Adams 2014** [91]	1	1	1	0	1	1	1	0	0	0	1	**7**
**Wegmann 2012** [92]	1	0	1	1	1	1	0	0	0	1	1	**7**
**Alhadad 2019** [93]	1	0	1	1	0	1	1	0	0	0	1	**6**
**Martin 2019** [94]	1	1	1	0	0	1	1	1	0	0	0	**6**
**Junge 2016** [95]	1	1	0	1	0	1	0	0	0	1	1	**6**
**Walker 2016** [96]	1	1	1	0	0	1	1	0	0	0	1	**6**
**Ross 2013** [97]	1	0	0	1	1	1	0	0	1	0	1	**6**
**Wittbrodt 2018** [98]	1	0	1	0	0	1	0	0	0	1	1	**5**
**Watson 2019**	1	0	1	1	0	0	0	0	0	0	0	**3**
**Heathcote 2018**	1	0	1	0	0	1	0	0	0	0	0	**3**
**Roberge 2011**	1	0	0	0	1	0	0	0	0	0	1	**3**

*AMSTAR* Assessment of multiple systematic reviews [99]. Q1 Was an a priori design provided? Q2 Was there duplicate study selection and data extraction? Q3 Was a comprehensive literature search performed? Q4 Did the search cover unpublished literature? Q5 Was a list of included and excluded studies provided? Q6 Were the characteristics of the included studies provided? Q8 Was the scientific quality used appropriately in formulating conclusions? Q9 Were the methods used to combine findings of studies appropriate? Q10 Was the likelihood of publication bias assessed? Q11 Were potential conflicts of interest listed?

**Table 2:** Study characteristics

**Author (year)**	**Aim**	**Number of studies**	**Number of participants**	**Cooling Interventions**	**Study context**	**Type of analysis**
**Adams 2014** [91]	Evaluate the change in heart rate for every 1% loss in body mass	20	^a^188 (173 M, 15 F)	Hydration	Athletic performance	ES + MR
**Alhadad 2019** [93]	Evaluate heat mitigation strategies to lower core temperature pre, mid and post exercise	123	^a^1470 (1382 M, 88 F)	Aerobic fitness, heat acclimation (low and high humidity), fluid ingestion (ad libitum, low and high consumption, hypohydrated, euhydrated), pre-cooling (cold water immersion, cold air exposure, cooling vests, cold fluid ingestion, ice slurry ingestion)	Athletic performance	ES
**Best 2018** [80]	Meta-analyze the effect of internal and external cooling methods applied before and during exercise on performance	10	^a^101 (101 M, 0 F)	Ice slurry ingestion, ice towels, oral rehydration, water dousing, external menthol application, cold fluids with menthol	Athletic performance	ES
**Bongers 2015** [89]	Evaluate precooling and percooling on exercise performance and thermoregulatory responses	28	^a^261 (283 M, 8 F)	Cooling vest, cold water immersion, cold water ingestion, cooling packs, mixed methods	Athletic performance	ES + MR
**Born 2013** [88]	Summarize evidence concerning compression garments on performance and recovery	^b^3	30 (30 M, 0 F)	Compression garments	Athletic performance	ES
**Brearley 2015** [81]	Efficacy of water immersion in firefighting settings to rapidly reduce core temperature	43	642 (517 M, 125 F)	Limb immersion	Firefighters	QUAL
**Burdon 2010** [86]	Determine whether ingesting cool beverages lowers core temperature and improves athletic performance	10	86 (86 M, 0 F)	Cold fluid ingestion	Athletic performance	ES
**Burdon 2012** [84]	Effect of beverage temperature on drink palatability	11	233 (208 M, 25 F)	Fluid ingestion	Athletic performance	ES
**Chalmers 2014** [65]	Investigate the effect of short term heat acclimation on physical performance	8	98 (79 M, 19 F)	Short term heat acclimation	Athletic performance	QUAL
**Chan 2015** [73]	Determine the effectiveness of multiple microenvironment cooling systems on physical performance	32	235 (231 M, 4 F)	Phase change garments, air cooled garments, cold-air cooled garments, liquid cooled garments, hybrid cooling garments, local cooling packs, cooling packs on neck and head	Sport, military, chemical protection, firefighting, and occupational health	ES + MR
**Choo 2018** [79]	Effect of cold water immersion and ingestion on psychophysiological and athletic performance	22	245 (213 M, 32 F)	Cold water immersion, ice slurry ingestion	Athletic performance	ES
**Daanen 2018** [64]	Systematically review and analyze heat acclimation decay and reacclimation	21	449 (428 M, 21 F)	Heat acclimation (decay)	Athletic performance	MR
**Douzi 2019** [75]	Evaluate effect of various methods of percooling on aerobic and anaerobic performance	36	^a,c^379 (356 M, 23 F)	Neck cooling, air-ventilation, cold fluid ingestion, ice vests, cooling garments	Athletic performance	ES
**Goodman 2019** [70]	Meta-analyze the effect of active hypohydration on cognitive performance	10	179 (124 M, 55 F)	Hypohydration	Cognitive performance	ES
**Goulet 2011** [85]	Effect of exercise induced dehydration on time trial performance	5	39 (32 M, 7 F)	Exercise induced dehydration	Athletic performance	ES + MR
**Hefferman 2019**	Systematically review trace mineral element supplementation on athletic performance	^e^15	^a^377 (328 M, 49 F)	Sodium supplementation	Athletic performance	QUAL
**Holland 2017** [67]	Determine effect of fluid ingestion on exercise performance	9	71 (64 M, 7 F)	Fluid ingestion	Athletic performance	ES + MR
**Jeffries 2019** [77]	Meta-analyze the effect of menthol use on athletic performance and psychophysiological responses	11	126 (126 M, 0 F)	Internal and external menthol application	Athletic performance	ES
**Jones 2012** [66]	Summarize the effectiveness of pre cooling methods	13	119 (108 M, 11 F)	Cold water immersion, cooling garment, cold water ingestion, ice slurry ingestion, leg cooling	Athletic performance	ES
**Junge 2016** [95]	The effect of environmental heat stress factors on cycling time trial performance	14	^a^145 (131 M, 14 F)	Environmental conditioning	Athletic and occupaltional performance	MR
**Martin 2019** [94]	Environmental heat stress on cognitive and military task performance	^f^31	^a^683 (563 M, 120F)	Environmental heat stress	Cognitive and military task performance	QUAL
**McCartney 2017** [68]	Fluid intake following dehydration on physical and cognitive performance	64	643 (598 M, 45 F)	Fluid ingestion	Athletic performance	ES
**McDermott 2009** [88]	Evaluate the effect of whole-body methods for reducing exercise induced hyperthermia	7	^a^68 (56 M, 12 F)	Cold water immersion, wet towels, cold air exposure, skin wetting with fan use, ice packs,	Athletic performance	QUAL
**McEntire 2013** [83]	Systematically review cooling techniques and practices among firefighters	27	^a^308 (287 M, 21 F)	Passive cooling in air, hand and arm immersion, foot immersion, cooling vests, hand cooling, intravenous cooling, fanning, fanning with misting, cooling sock, liquid and air cooled suits,	Firefighters	QUAL
**Rahimi 2019** [78]	Heat acclimation on athletic performance and psychophysiological responses	11	215 (195 M, 20 F)	Heat acclimation	Athletic performance	ES
**Ranalli 2010** [74]	Effect of body cooling on exercise performance	^c^9	^a^107 (92 M, 15 f)	Ice vest, cold water immersion, cooling collar	Athletic performance	%Increase
**Ross 2013** [97]	Evaluate the established pre-cooling literature	64	631 (587 M, 44 F)	Cold air, cold water immersion, cooling packs, cooling vests, ice towels, cold water ingestion, cold air inhalatiion	Athletic performance	QUAL
**Ruddock 2017** [72]	Does cooling during exercise decrease physiological strain and improve exercise performance	15	135 (135 M, 0 F)	Cold fluid, ice slurry, neck cooling collar, hand cooling,	Athletic performance	ES
**Stearns 2010** [87]	Meta-analyze the effect of protein and carbohydrate ingestion on exercise performance	^d^1	^a^13 (7 M, 6 F)	Protein ingestion	Athletic performance	QUAL
**Tomes 2017** [71]	Effect of wearing protective body armour	16	433 (353 M, 80 F)	Clothing variety (protective)	Law enforcement personnel wellbeing	QUAL
**Tyler 2015** [90]	Effect of pre and percooling on exercise performance and capacity in the heat	38	335 (318 M, 17 F)	Mist and fan, ice vest, neck cooling collar	Athletic performance	ES
**Tyler 2016** [69]	Effect of heat acclimation on physiological, perceptual and performance variables	96	1056 (980 M, 76 F)	Heat acclimation	Athletic performance	ES
**Vandenbogaerde 2011**	Conduct a meta-analysis to determine the effect of carbohydrates on athletic performance	154	1628 (1469 M, 159 F)	Carbohydrate ingestion	Athletic performance	MR
**Walker 2016** [96]	Investigate the effect of fighting fires on hydration status	10	225 (214 M, 11 F)	Hydration	Firefighter hydration	QUAL
**Wegmann 2012** [92]	Effect of pre cooling on sport performance	27	268 (260 M, 8 F)	Cooling vest, cold water immersion, skin wetting, cooling packs, cold water ingestion, cold air	Athletic performance	ES
**Wittbrodt 2018** [98]	Determine the impact of dehydration on cognitive performance	33	463 (396 M, 67 F)	Hydration	Athletic and occupational cognitive performance	ES + MR

*M* Male; *F* Female; *ES* Effect size; *MR* Meta-regression; *QUAL* Qualitative analysis

^a^ Distribution of male and female participants determined from the original investigations

^b^ Only includes studies examining the effect of core temperature as exercise performance was conducted in temperate conditions

^c^ Includes only the studies examining effect on aerobic performance/ excludes anaerobic performance

^d^ While this review examined and meta-analysed the results of 11 studies, they marked that protein ingestion was only

^e^ Number of studies pertaining to sodium ingestion only

^f^ Number of studies pertaining to the heat only

### Quality assessment and review characteristics

The quality assessment of the 39 systematic reviews meeting our inclusion criteria using the AMSTAR checklist are displayed in Table 1. Of the reviews evaluated, 11 were classified as “high quality”, 25 were classified as “moderate quality” and the three reviews classified as low quality were excluded from further analysis. The mean quality score was 8 ± 2.

The characteristics of the 36 reviews fulfilling the selection criteria and quality assessment are displayed in Table 2. In total, the 36 reviews were comprised of 1047 studies (including duplicates), equating to an average of 27 studies per review (median: 16). There was a total of 12,684 participants (average: 12 per study), comprised of 11,510 (91%) males and 1204 (9%) females. The most commonly investigated intervention was cold water immersion (10 reviews); followed by hydration maintenance and cold fluid/ice slurry ingestion (9 reviews); cooling vests and environmental conditioning (mentioned in 7 reviews); cooling packs (6 reviews); skin wetting and heat acclimation (5 reviews); ice towels, fanning, neck cooling collars (3 reviews), (ambient or cold) air cooled garments and liquid cooled garments, external menthol application, internal menthol use and hand cooling (2 reviews); and finally, mixed method cooling, compression garments, intravenous cooling, cold air inhalation, protein and carbohydrate ingestion, protective clothing, hybrid cooling clothing, aerobic fitness, and sodium supplementation (1 review each). Of the 36 reviews, 29 focused solely on physical performance, five focused on occupational health and/or performance, and three contained aspects of both fields, and one focused solely on cognitive performance. Finally, 17 of the systematic reviews only contained traditional Hedge’s g type effect size comparisons, 10 solely contained qualitative analyses, six contained both Hedge’s g and meta-regression analysis, three used meta-regression only, and two used alternative forms of comparison (one, absolute mean difference; the other, percent increase in performance).

### List of additionally identified interventions

In addition to the interventions identified through the systematic reviews, original research articles on seven additional interventions, namely: providing shading [24, 100], improving clothing design [21, 49], utilizing clothing with ventilator incorporated into the clothing [101, 102], electrolyte consumption [103, 104], taking intermittent rest breaks [105], and slowing pace/reducing work intensity [106].

### Effectiveness of interventions

**Fig. 2:**
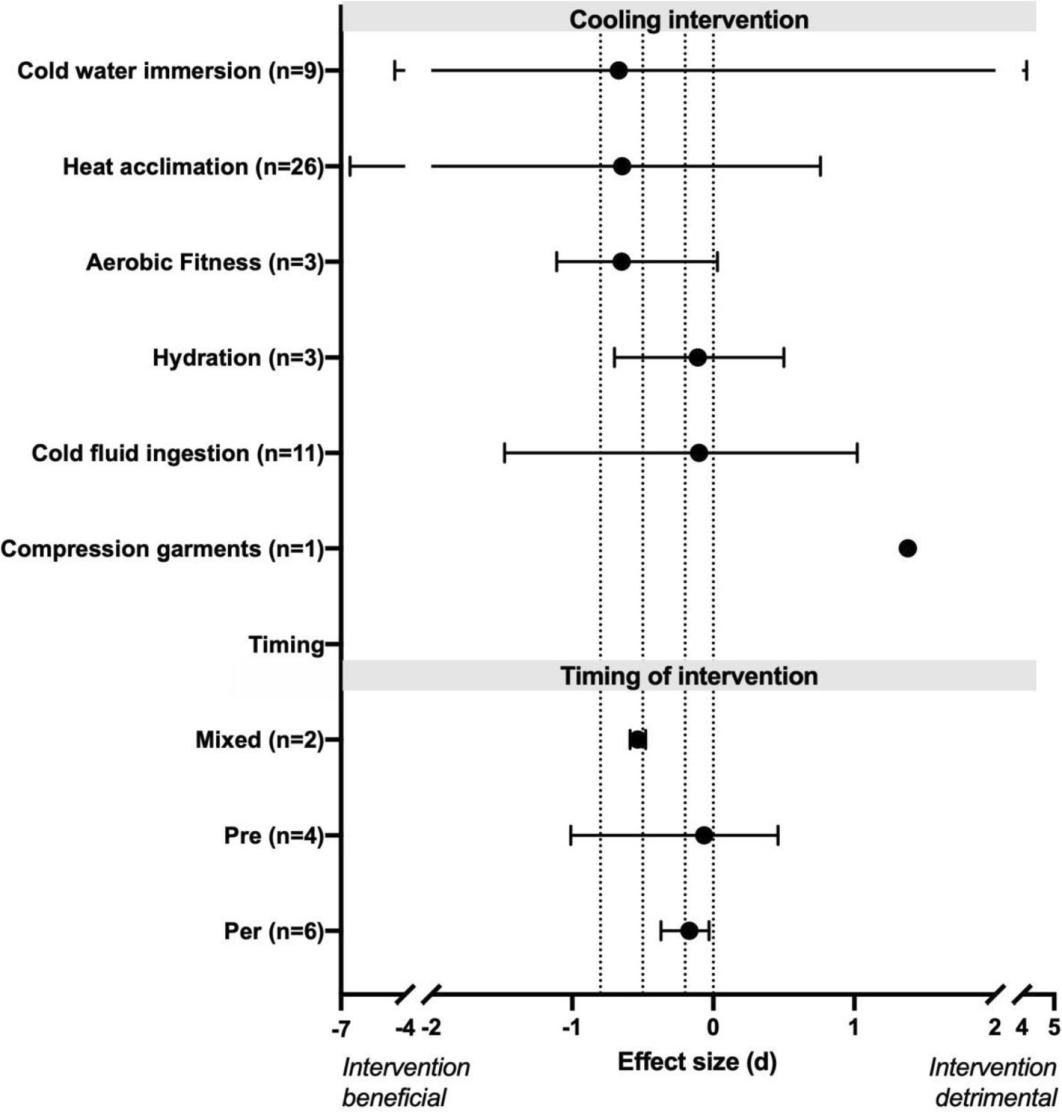
Median (range) effect of type and timing of cooling intervention on physiological (pooled heart rate and core temperature) from systematic reviews included in the umbrella review. Number inside of bracket on y-axis denotes the number of effect size comparisons per intervention. Dashed upright lines denote threshold of no (0), small (0.2), moderate (0.5) and large (0.8) effect sizes

**Fig. 3:**
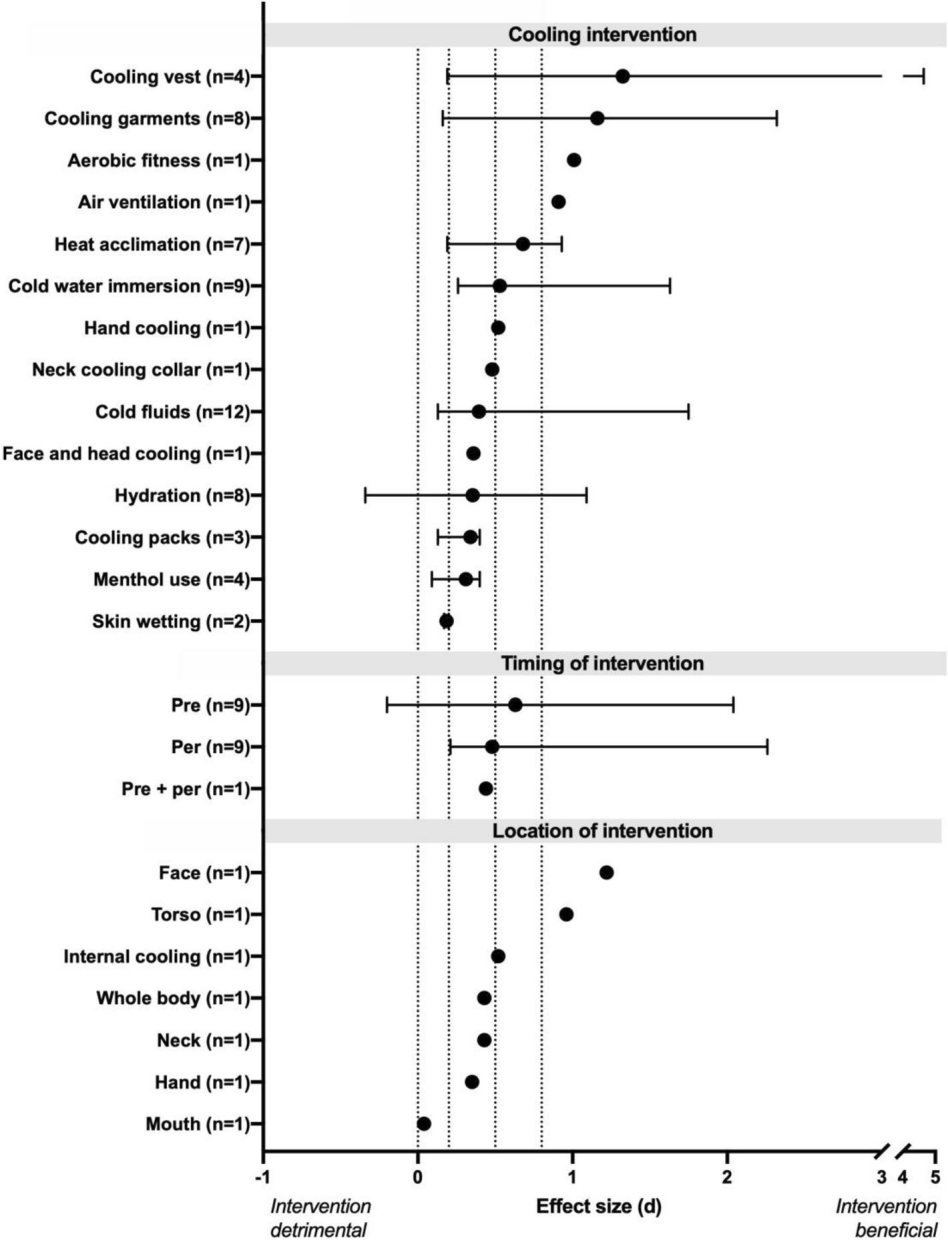
Median (range) effect of type, timing and location of cooling intervention on physical performance from systematic reviews included in the umbrella review. Number inside of bracket on y-axis denotes the number of effect size comparisons per intervention. Dashed upright lines denote threshold of no (0), small (0.2), moderate (0.5) and large (0.8) effect sizes

Overall, from the 36 systematic reviews that met our criteria, 22 contained Hedge’s g type meta-analyses. From these 22 reviews, 63 comparisons pertaining to physiological responses (Additional file 1: Appendix 2; Fig. 2), 84 comparisons pertaining to all physical performance (Additional file 1: Appendix 3; Fig. 3), 10 comparisons regarding cognitive performance (Additional file 1: Appendix 4) and 23 comparisons regarding perceptual responses (Additional file 1: Appendix 5). To improve clarity, the interventions were grouped into relevant categories of environmental conditioning, clothing, personal cooling, physiological conditioning, pacing and hydration and nutrition. Comparisons of timing of intervention, location of intervention and mixed interventions were further grouped together.

#### Physiological responses

All of the individual effect size values from the included reviews pertaining to the physiological responses, are displayed in Additional file 1: Appendix 2 and the median and range of the reported effect sizes separated by intervention are displayed in Fig. 2. On average, the most effective intervention for reducing pooled physiological strain was heat acclimation − 0.83 (median: − 0.65, min: − 6.57 [maximum heart rate], max: + 0.76 [mean heart rate during time trial]). This was followed by improving aerobic fitness (mean: − 0.58, median: − 0.65, min: − 1.11 [max core temperature], max: + 0.03 [change in core temperature from baseline]), cold water immersion (mean:-0.32, median: − 0.67, min: − 4.48 [reduced skin temperature at baseline], max: + 4.15 [change in skin temperature from baseline]), maintaining hydration status (mean: − 0.10, median: − 0.11, min: − 0.70 [change in core temperature from baseline], max: + 0.50 [maximum core temperature]), cold fluid ingestion (mean: − 0.06, median: − 0.01, min: (− 1.48 [rectal temperature at rest], max: + 1.02 [change in core temperature from baseline]),while compression garments were found to be ineffective/detrimental (increase in mean core temperature of + 1.38).

Additionally, per-cooling (i.e. cooling applied during work/exercise) provided a mean score of − 0.18 (median: − 0.17, min: − 0.37 [rectal temperature], max: − 0.03 [mean heart rate]) and was equally effective for lowering physiological strain as pre cooling (mean: − 0.17, median: − 0.07, min: − 1.01 [core temperature], max: + 0.46 [change in core temperature]. Further, combining cooling methods proved effective for lowering core temperature (− 0.59).

#### Physical performance

All of the individual effect size values from the included reviews pertaining to physical performance, are displayed in Additional file 1: Appendix 3 and the median and range of the reported effect sizes separated by intervention are displayed in Fig. 3. The most effective methods proved to be cooling garments, led by liquid (+ 1.86), then air (+ 1.76) and hybrid (+ 1.61) cooling garments, with cooling vests ranking lower but still highly effective (+ 0.73). Following were improvements of physiological adaptations such as physical fitness (+ 1.01) and heat acclimation (+ 0.65), as well as facilitating natural heat loss through improved ventilation (+ 0.91). Next were the array of personal cooling methods, however, these were highly variable within method. In this category, cold-water immersion was most effective (+ 0.53), followed by hand cooling (+ 0.52), neck cooling collars (+ 0.48), cold water ingestion (+ 0.40), cooling pack (+ 0.34), menthol (+ 0.31) and skin wetting (+ 0.19). Maintaining hydration status (+ 0.35) and mixed method cooling (+ 0.49) were both found to be moderately effective as well.

For timing of cooling intervention application, precooling (+ 0.63) was found to be more effective than per cooling (+ 0.48) as well as pre and per cooling (+ 0.44). Additionally, face cooling (+ 1.22) was most effective followed by cooling the torso (+ 0.96), internal (+ 0.52), neck (+ 0.43), whole-body (+ 0.43), face and head (+ 0.36), hand (+ 0.35) and mouth (+ 0.04) cooling.

#### Cognitive performance

To date, only the effect of maintaining hydration status on cognitive performance has been systematically investigated, with the available scores displayed in Additional file 1: Appendix 4. Of the eight values given between the two review, on average, dehydration caused a moderate decrease in cognitive performance (mean: − 0.56, median: − 0.27, min: − 2.61 [overall cognition], max: − 0.1 [reaction time]).

#### Perceptual responses

The reported perceptual scores are displayed in Additional file 1: Appendix 5. Heat acclimation was most effective at reducing ratings of perceived exertion (− 0.63), followed by water dousing (− 0.50), cold fluid ingestion (mean/median: − 0.21), and cold water immersion (− 0.14). Menthol application was most useful for reducing thermal sensation (− 0.54), as was heat acclimation (− 0.53), followed by cold water immersion (− 0.50) and cold fluid ingestion (− 0.20). Additionally, skin wetting was reported to be very effective for improving thermal comfort (− 1.35). Pre-cooling was reported to slightly increase subsequent exercise ratings of perceived exertion (+ 0.17), whereas per cooling reduced ratings of perceived exertion (− 0.44), thermal sensation (− 0.64) and improve thermal comfort (+ 1.29). Finally, cooling before and during exercise was reported to reduce ratings of perceived exertion (− 0.48).

### Qualitative reviews and reviews containing meta-regressions

In addition to the interventions that are identified in the previous sections and in Additional file 1: Appendix 2, 3 and 4, other interventions identified in four of the qualitative reviews were: intravenous cooling and misting fans [83], cold air inhalation and ice towels [97], protein ingestion [87], sodium supplementation [76] and variants of protective clothing [71]. The qualitative reviews provided further discussion concerning limb immersion for firefighters [81], the benefits of short term heat acclimation for athletic performance [65], the impact of environmental stress on military cognitive and other task performance [94], effect of whole-body cooling methods for reducing exercise hyperthermia [88], and the effects of fighting fires on hydration status [96]. Further one review [74] provided mean percent-changes in athletic performance with ice vest, cold water immersion and cooling collars, but contained no additional meta-analyses.

In total, nine reviews contained meta-regression analyses: four on hydration [67, 85, 91, 98], and one each for external and internal cooling strategies [89], clothing [73], deacclimation and reacclimation [64], environmental control [95] and carbohydrate ingestion [107].

### Evaluation of interventions for strength of evidence, cost, feasibility, and environmental sustainability

An overall presentation of the interventional analysis can be found in Table 3. Collectively, interventions that focused on cooling the environment were most effective as well as most feasible in indoor environments. However, the effectiveness and feasibility of these interventions were highly variable due to the infeasibility of attempting to cool outdoor or large indoor spaces. In general, these interventions were also expensive and imposed a large environmental burden, with the exception of providing shading.

**Table 3:** Complete list of interventions evaluated by effectiveness, cost, feasibility and sustainability

Intervention	Strength of evidence	Effectiveness (best/ (worst) scenario)	Cost (considering acquisition and operation)	Feasibility (indoor/ small spaces	Feasibility (outdoor/ open spaces)	Environmental impact (ecological foot print)
Environmental conditioning						
Air conditioning	2	**3.0 /** (0.75)	HIGH	−0.8	−2.9	HIGH
Misting fan	2	**2.5** / (1.4)	MOD	−1	−1.8	MOD
Fanning	3	**2.4** / (1.2)	MOD	−0.8	−1.9	MOD
Shading	1	**2.3 /** (0.6)	LOW	−0.7	−1.3	LOW
Clothing						
Air cooled garments	3	**3.0** / (2.3)	HIGH	−2	− 2.6	HIGH
Liquid cooled garments	3	**3.0** / (2.3)	HIGH	−2.3	−3	HIGH
Cooling vest	3	**1.7** / (1.7)	MOD	−1.3	−2	MOD
Elevated design clothing	1	**1.2** / (1.2)	MOD	−1.4	− 1.7	MOD
Ventilator-incorporated clothing	1	**1.1** / (1.1)	MOD	−1.8	− 1.8	MOD
Compression garments	3	**1.0** / (0.8)	MOD	−1.3	−1.6	LOW
Protective clothing	2	**0.7** / (0.6)	MOD	−1.6	− 1.7	LOW
Innate conditioning						
Short term HA	3	**1.9** / (1.8)	NONE	−1.7	−1	NONE
Medium term HA	3	**2.5** / (2.4)	NONE	−1.4	−0.9	NONE
Long term HA	3	**2.9** / (2.6)	NONE	−1.6	− 1.1	NONE
Aerobic fitness	3	**2.9** / (2.1)	NONE	−1.2	−1.0	NONE
Personal cooling solutions						
Cold water immersion	3	**2.2** / (1.9)	MOD	−1.6	−1.9	LOW
Intravenous cooling	2	**2.3** / (1.7)	HIGH	−2.3	−2.6	MOD
Cold air inhalation	2	**2.1** / (1.2)	HIGH	−2.1	−2.5	MOD
Cold fluid ingestion	3	**2.0** / (1.7)	MOD	−1.3	−1.5	LOW
Cooling packs	3	**1.8** / (1.5)	MOD	−1.6	−2.1	MOD
Skin wetting	3	**1.7** / (1.4)	LOW	−1.9	−1.6	LOW
Neck/head cooling	3	**1.2** / (1.1)	MOD	−1.7	− 1.5	LOW
Ice towels	2	**1.5** / (1.3)	MOD	−2.2	−1.9	LOW
Cooling glove	2	**0.8** / (0.6)	MOD	−1.8	−2.2	MOD
External menthol use	3	**0.7** / (0.3)	LOW	−2	−1.9	LOW
CInternal menthol use	3	**0.7** / (0.3)	LOW	−1.8	− 1.7	LOW
Pacing						
Change in work schedule	0	**2.0** / (1.5)	NONE	−1.5	−1.6	NONE
Work intensity reduction	1	**1.8** / (1.8)	NONE	−1	−1.1	NONE
Breaks	1	**1.3** / (1.3)	NONE	−1	−1.1	NONE
Hydration and nutrition						
Hydration	3	**2.0** / (2.0)	LOW	−1	−0.9	NONE
Electrolyte consumption	1	**1.2** / (0.9)	LOW	−0.7	−0.6	NONE
Carbohydrate ingestion	2	**0.6** / (0.6)	LOW	−0.7	−0.6	LOW
Protein ingestion	2	**0.3** / (0.3)	LOW	−0.7	− 0.6	LOW

Table 3 is a summary table of all the identified available solutions to mitigate occupational heat strain, which have been evaluated on four-point scales for five different criteria: the strength of evidence in the literature, the proven effectiveness of the method in best (bolded numbers) and worst case (numbers in parentheses) scenarios, the cost, the feasibility (separated into indoors and outdoors) and the environmental impact. Scores for strength of evidence were: 0, expert knowledge or non-human based research; 1, original research; 2, systematically reviewed but not meta-analysed; and 3, systematically reviewed and meta-analysed. Effectiveness scores were: 0, ineffective or detrimental; 1, slightly beneficial; 2, moderately beneficial; and 3, beneficial. Cost evaluations were: none, low, moderate (MOD) and high. Feasibility scores were: 0, no disruptions to normal work; − 1, minor interruptions; −2, moderate interruptions; and − 3, major interruptions. Environmental impact scores were: none, low, moderate (MOD) and high.

Both air and liquid-cooled clothes were highly effective but also generally infeasible, costly and imposed a large environmental burden. Improving the design of clothing had a small beneficial effect, moderate cost and environmental impact, but generally scored highly feasible. Additionally, cooling vests and packs ranked as moderate across the board.

Methods focusing on improving physiological conditioning, including both physical fitness as well as heat acclimatization were found to be the next most effective group. These types of interventions were consistently effective in all environments, pose no additional costs to the companies and have no environmental impact.

Personal cooling methods scored as less effective than environmental conditioning, but were less variable, less costly and had a lower environmental impact. In particular, cold-water immersion was most effective, as well as moderately costly and feasible, with a low environmental impact. All internal cooling methods were ranked as moderately effective, however, intravenous and cold air inhalation were scored as expensive, infeasible, and moderately detrimental for the environment, whereas cold fluid ingestion was only moderately expensive, feasible and had a low environmental impact. Improving hydration had a consistent moderate effect, was inexpensive, feasible and did not impose an environmental impact. Finally, both forms of sensation manipulation were scored as generally ineffective, albeit not costly or harmful towards the environment.

Pacing strategies had the lowest strength of evidence, as none of these interventions have currently been systematically analysed. However, data from original research and modelling studies suggest that these types of interventions may be moderately beneficial, effective in both indoor and outdoor scenarios, are low cost, highly feasible and cause no additional environmental harm.

Hydration is ranked as moderately effective for maintaining work performance. It is low cost, highly feasible in most scenarios (more difficult outdoors) and poses no additional environmental impact. Electrolyte supplementation, combined with proper hydration is a feasible mildly effective way of mitigating heat stress. Both protein and carbohydrate consumption were ranked as low cost, feasible and having a low environmental impact, but were scored as ineffective.

## Discussion

### Principal findings

This review was the first to both systematically review all available cooling interventions for improving worker health and productivity in hot occupational environments and evaluate these interventions based on their cost, feasibility, transferability and environmental impact. From our results, a clear hierarchy of effectiveness was observed, wherein environmental conditioning was most effective, followed by cooling garments, state of physiological adaptation, and personal cooling interventions. However, taking into account the cost, transferability, feasibility and environmental sustainability of the interventions, the interventions that were most effective at improving health and performance were typically the most expensive, least feasible and were not generally adaptable to multiple types of environments. Accordingly, “the best intervention” to use, will be unique to the individual user depending on the given situation. Below, we discuss considerations for the different interventions to help practitioners identify the best cooling solution for their particular needs.

### Environmental conditioning

Removing the environmental stress with complete cooling (air-conditioning) is clearly the most effective method for alleviating the detrimental effects of occupational heat strain on physiological strain and work performance, as it effectively removes the environmental source of heat stress. Air-conditioning is, however, highly energy intensive, thereby incurring a significant economic cost [108], greenhouse gas emissions when energy supplies comes from non-renewable sources which further contributes to pollution related fatalities [109], and directly worsens global warming and the urban heat island effect due to the hot exhaust air production [110]. Moreover, air-conditioning cannot be used outdoors, nor in large factory settings. It is possible, however, to improve the efficacy of air-conditioning by cooling small break rooms to provide workers reprieve from the heat at specific intervals. Further, as renewable sources of energy become more prevalent, this option will become increasingly attractive, however, the environmental impacts of the production and waste of this technology across its lifespan continues to have tremendous environmental impact regardless of energy source or efficiency [111].

Facilitated ventilation, e.g. through the use of electric fans presents an alternative method to alter the environmental settings to support convective and/or evaporative heat loss and lower occupational heat strain (Fig. 3) at a considerably lower operation and production cost compared to air conditioning. Further, electric fan use can be used in conjunction with air-conditioning to improve the efficacy of air-conditioning at higher temperatures, allowing for the power cost to be diminished [112]. Fanning is also more readily personalized by directing its flow towards specific workers, rather than cooling an entire area, can be transported to remote work sites, and can be used both during work (especially for less mobile tasks) and/or can be used at specific cooling areas where workers can take intermittent cooling breaks. Further, miniature electrical fans can be incorporated into chairs, for seated workers, to deliver effective cooling while minimizing disturbances to the work environment [113]. Of note, the efficacy of fan use is diminished when highly insulative (e.g. protective clothing) is worn or if work is undertaken in exceptionally hot and dry environments [114].

Although the effectiveness of shading has yet to be reviewed systematically, original investigations into reducing the solar (radiative) heat load on workers is promising, revealing that work capacity can be more than doubled with the removal of an external radiative load [24]. The primary benefit of shading is to reduce the added thermal load to outdoor workers caused by working in the sun; alternatively, radiation screens can be used within manufacturing shops to shield workers from radiation originating from hot machinery. Providing stationary shading outdoors, however, can be difficult as temporary shelters must be transported with the crews, which may prove especially difficult for workers (e.g. agricultural workers) who may spend most of the day on foot away from any permanent structures. However, the addition of shaded break areas have been demonstrated as an integral part of a heat health plan to improve worker performance in the agricultural sector [115]. Moreover, wearing wide brimmed hats and long loose fitting clothing is a more feasible method for reducing radiative heat loads as well as protect against UV radiation.

### Clothing optimization options

Following removing or improving the environmental heat stress, clothing options were the next most effective way for improving health and performance outcomes, with the exception of compression garments which were found to actually increase core temperature [82]. Often in manual labour occupations, personal protective equipment (commonly referred to as PPE) must be worn, which is highly insulative and impedes dry and evaporative heat loss by creating a microenvironment underneath the clothing which is even more thermally stressful than the environmental conditions [20, 21]. This has been extensively demonstrated in emergency response and chemical waste disposal workers, military and firefighters [71, 83, 94, 116] and has received some attention in the construction sector as well [73]. While potentially less studied (or entirely unstudied), protective clothing is often required in other major industries such as manufacturing and agriculture; however, manufacturing and agriculture were not represented in any of the reviews included in the present study, and construction was present in only one review. Accordingly, these fields require more investigation.

Contrary to the insulative effects of protective clothing, a microenvironment can be created which greatly favours heat loss, as demonstrated by the effectiveness of cooling garments to reduce physiological strain and improve work performance demonstrated in Figs. 2 and 3. Full-bodied garments typically consist of some sort of a suit that is either lined with tubing through which cooled water can be cycled through, or else a suit which allows for air (cooled or ambient temperature) to flow across the body and then out of the suit into the surrounding environment [73, 117]. Generally, these suits are not feasible for the vast majority of occupations, as they typically require to be fixed to a cooling source through tubes in order to pass the liquid or air cooling. Additionally, both air and water perfused suits are either economically and environmentally costly and/or difficult to implement. Improved models of these garments do exist, such as air-perfused suits or shirts, which may be less costly to operate compared to water perfusion suits due to the reduced weight and energy cost to operate (due both to lower cooling requirements and mechanical power needed to circulate air) and greater availability and mobility of the required equipment. Further, novel innovations to clothing are continuously being generated, as is the case with ventilator-incorporated clothing [102]. Here, a personal fan is embedded into the clothing, resulting in greater airflow across the skin surface facilitating heat loss through convection and evaporation. Additionally, newer models of water-perfused suits have been developed which improve upon the issue of portability, however, this typically results in the reduction of effectiveness [118].

A popular iteration of cooling garments are cooling vests, which come in different varieties but typically can be categorized into one of two forms (or a combination of the two): a conductive cooling variant in which a solid coolant (either a phase change gel or ice) is inserted into the vest [119] or else an evaporative cooling variant wherein the vest is composed of materials that can hold water and possess qualities that facilitate air flow and the primary cooling ability comes from the evaporation of this water. Cooling vests are beneficial as they can be worn less invasively under typical personal protective equipment and are less cumbersome and invasive during work. Conductive cooling vests are effective regardless of the environmental conditions but are especially effective in very humid environments where evaporative vests become less effective, or else underneath large amounts of insulative clothing where evaporative heat loss is minimized [116], and wet clothing is uncomfortable. On the other hand, conductive cooling vests lose effectiveness as the coolant melts, can also be heavy thereby increasing the endogenous heat production, and finally will reduce the effective skin surface area for evaporation. Further, cooling vests can range in price from $60–200 USD and could therefore be prohibitively expensive for workers in low resource settings and multiple vests per worker (for replacement purposes) were bought for large workforces. Moreover, cooling vests are largely composed of plastics and require equipment and energy usage to cool them; thereby imposing further environmental costs when the energy supply comes from non-renewable sources.

One aspect of clothing not systematically investigated by randomized control trials in ecological settings is the ability to improve the characteristics of the clothing itself, likely because clothing research is often performed on thermal manikins to determine how heat loss can be improved, rather than tested on humans in the laboratory or field, and therefore do not yield results that can be readily systematically analysed. Primary aspects of clothing that can be modified to improve heat loss is the fit (microclimate air gaps) and ventilation design (fabric air permeability and design of ventilation openings), with both facilitating larger air flow across the skin, when the worker is mobile or in ventilated areas, to take away heat and moisture [120]. Additionally, tighter fitting clothing results in increased feelings of clamminess and discomfort, and with fabric type and structure playing an important role [49]. However, results for different fabric types do not provide a single answer, as preference changes based on specific work conditions. Further, in some situations, the amount of clothing can be reduced to expose more skin directly to the air to improve heat loss. However, it is important to note that outdoors, the need for heat loss needs to be balanced with the need to protect the skin from solar radiation for both heat and UV protection [121]. Therefore, in outdoor occupations with solar radiation, hats and long, light, brightly coloured or reflective, loose fitting and breathable clothing should be worn. Finally, recent studies [122] have experimented with creating ventilation patches made of lighter, more breathable materials, in areas of the work suits that are naturally more protected (such as in the groin, lower back, arm pit and behind the knees) and have demonstrated that even minor improvements such as these can help improve heat loss to the environment.

### State of physiological adaptation

Following the removal or mitigation of the environmental and/or microclimatic conditions, improving the physiological conditioning of the workers through (physiological) heat acclimatization or physical fitness was the next best type of intervention [69, 78, 93]. Heat acclimatization, within this context, refers to specific physiological adaptations (i.e. lowering of core temperature and heart rate, increased plasma volume and sweat rate, as well as improved thermal comfort in the heat) that occur as a result of prolonged exposure to heat stress [69], and not behavioural adjustments to cope with the heat. This method was highly effective at improving health and productivity outcomes, and even short term heat acclimatization (less than 7 days) was shown to have a moderately strong effect at improving physical work performance in the heat (effect size of 0.52), whereas long term heat acclimatization (14 days or longer) was one of the overall most potent methods to improve performance in the heat (effect size of 0.93). Additionally, heat acclimatization is free, occurs naturally and poses no additional stress on the environment.

It must be noted that the original heat acclimatization investigations informing these meta-analyses were typically conducted during physical performance in controlled environmental chambers at temperatures which may exceed what would be observed in the field. However, the protocols in these original investigations are typically much shorter than the average workday (1–2 h in duration). A further consideration is that the acclimatization process may be hindered when non-active time is spent in air-conditioned environments [123]. Accordingly, how these findings translate to real-life occupational scenarios is unknown, and therefore should be considered with caution and further investigated. There is some support for the transferability of these results, as occupational epidemiological studies have shown workers who are unacclimatized (less than 2 weeks on the job) comprise more than 75% of occupational heat related deaths [124]; however, how much of this discrepancy is due to physiological verses behavioural adaptations remains unclear.

Similar to heat acclimatization, physical conditioning (i.e. improving the aerobic fitness of the workers) may indeed improve heat tolerance and lower occupational heat strain [93]. However, physical conditioning through training interventions may be considerably more arduous to achieve and hence less feasible to implement; although it would provide parallel (general) health benefits for workers with low cardiorespiratory fitness levels [125]. While further research is needed to examine how to best incorporate this information into occupational heat strain recommendations, improving health, comfort, and productivity in the heat could be listed as additional reasons for active living promotion. Additionally, this information is in-line with previous research regarding which workers are at greater risk for heat illness [126]. Further, previous research has demonstrated that physical fitness mitigates the age-induced decline in thermoregulatory capabilities, and therefore should be particularly encouraged amongst aged workers [127].

### Personal cooling options

One notable attribute of the personal cooling interventions assessed was that, overall, the reported effect sizes were far more variable than other interventions. This is likely because the effectiveness of these types of cooling interventions will be environment dependent, as has been previously illustrated [32]. Of all the external cooling strategies, cold water immersion was the most effective cooling intervention, primarily during precooling but during intermittent cooling as well [66, 75, 79, 89]. Cold water immersion is most effective when cold water is used and the entire body is submerged; this practice, however, is entirely implausible for nearly all occupational settings and likely is only realistic within a sporting context. Alternatively, submerging the forearms has been demonstrated to be highly effective at lowering core temperature during relatively short breaks and is often employed by firefighters [128, 129]. Benefits of cold water immersion are that it will be effective, regardless of the prevailing environmental conditions as the cold water acts as a heat sink, removing heat from the workers’ bodies via conduction. The feasibility of cold water immersion, even if just to the forearms, is somewhat limited as this cooling intervention can only be applied before the work shift and during intermittent breaks, and further, fine motor control is impaired following immersion [130]. Moreover, the colder the water used for submersion, the faster the rates of cooling will be, necessitating less break time to recover [131]; however cooling the water to greater extents, especially in hot environments, necessitates greater equipment and electrical consumption for cooling. To address these feasibility and cost issues, adequate rest stations can be established where multiple workers are able to cool concurrently, and based on the strength of the cooling effect and that meaningful cooling occurs at as little as 20–22 °C water temperature [131, 132], this may be the optimal method for delivering as much cooling as possible during short periods of time.

Following cold-water immersion, cold fluid ingestion, and particularly crushed ice/ice slurry/ice slushy ingestion, was the most studied personal cooling method, but the effect for performance was highly variable [66, 79, 80, 86, 89, 92] and the effect for mitigating physiological strain was modest [79, 80, 86]. Cold fluid ingestion is effective by working as a heat sink, removing heat from the body to warm the fluid [133]. Contrary to some conventional wisdom, this process is not harmful to the body and will not result in an elevated metabolic rate in hot environments [134]. Ice slurry ingestion has been suggested to be most effective at rest, as well as in hot-humid conditions, due to corresponding reductions in sweating when consumed during exercise [135]. Cold fluid ingestion, relative to external cooling methods, has the added benefit of helping to maintain hydration status, however, studies have demonstrated that individuals will drink less ice slurry compared to cool water [136], and therefore ice slurries should not be relied upon as the sole hydration source. Indeed, one review included in our analysis indicated the ideal drinking temperature for palatability and therefore replacing fluid loss, is 10 °C [84].

The use of cooling packs, cooling collars and wet towels containing ice were other, albeit less effective, cooling options [73, 75, 89]. This smaller cooling effect, relative to cold water immersion or cooled garments, is likely due to the smaller surface area for cooling of the packs, and the effect appears to be generally uniform regardless of the area of the body applied to (i.e. hands, neck, face and head; Fig. 3). Due to the areas of application, cooling packs may interfere with the workers performing their tasks and are likely only useful during breaks. Cooling packs typically are not inexpensive to purchase, are largely composed of plastics and also require the equipment and energy usage required to cool them; thereby imposing further environmental costs when the energy supply comes from non-renewable sources. Ice towels, consisting of wet towels wrapped around crushed ice, which are then then draped around the neck of the wearer, are a lower cost alternative to more expensive phase change materials [137, 138]. However, this method requires time to prepare the towels, is likely infeasible for workers having to wear bulky clothing which would become heavy and uncomfortable when wet, and requires accompanying equipment to produce and/or keep ice cold. Vacuum gloves, consisting of an active cooling component and a negative pressure system, were developed in an attempt to counteract the cold-induced vasoconstriction that occurs when the hands are exposed to a cool stimulus. This rationale has been disproven, however, as cold-induced vasoconstriction in the hand does not occur in people with elevated core temperature temperatures, irrespective of hand temperature [129], and thus this expensive and energy intensive intervention should be discounted.

Skin wetting (Fig. 3) only provided moderate improvements of work performance [80] and no systematic reviews evaluated its effectiveness at lowering physiological strain. However, skin wetting requires only water and therefore is readily available for use at no additional cost to employers (assuming they have access to running water) and generally poses little to no additional strain on the environment, except in areas with shortages of available drinking water [139]. In such scenarios, however, skin wetting has the added benefit of being able to use water that may not be of a sufficient quality to drink. This aspect of skin wetting may be particularly beneficial as skin wetting has been shown to reduce natural sweat rate without incurring elevated core temperatures or heart rate [132], thereby providing a method of slowing the dehydration process when drinking water is limited. One drawback to this method is that its use is not always feasible, especially indoors and/or when large amounts of personal protective equipment are worn.

Finally, the application of menthol gels to the skin or adding menthol salts to water that is either swilled in the mouth or ingested was one avenue of modestly improving work performance, but not improving health outcomes [77]. Menthol is moderately beneficial at improving thermal comfort and thermal sensation (Additional file 1: Appendix 5) but, as it is not associated with actual body cooling, may in fact be detrimental to workers’ health. Specifically, workers who feel cooler may work harder, thereby producing more heat without any meaningful additional cooling provided by menthol, resulting in greater heat storage within the body, leading to at greater risk for heat illness. Accordingly, this method is not recommended.

### Pacing strategies

A potential intervention to minimize the effect of occupational heat strain that was absent from all systematic reviews was the use of pacing strategies. These methods are among the simplest, least costly and environmentally friendly strategies as they simply consist of slowing down or ceasing to work. Recent work using time-motion analysis has demonstrated that workers will take more unplanned breaks as environmental temperatures rise [140]. While this method is likely beneficial for health purposes, it will diminish work productivity and may therefore be discouraged by employers [17]. Decreasing the intensity at which people work has similar effects to taking unplanned breaks [106], but this may have additional negative health consequences for the workers, due to decreased pay in regions that operate on a piece rate pay scheme [141].

One intervention identified by the secondary search was to change daily working hours [100, 142]. By shifting the workday forward (depending on the job and the start time), the mean ambient temperature and solar radiation throughout the workday would be lower. Although cost efficient and easily implementable for some, the workday would still encompass the hottest hours of the day [100, 142], and in some tropical countries, even a 3 h earlier work shift start would be insufficient to prevent heat-induced labour losses due to planned or unplanned breaks [140, 142, 143]. Alternatively, the workday can be divided into two shifts while taking a break or “siesta” in the middle of the day in order to avoid peak temperatures [144]. However, this strategy is invasive towards the workers’ personal lives, and with workers living increasingly further from their places of work [145], and therefore longer commute times, this option is becoming increasingly less feasible. Although the effectiveness shifting work hours has been modelled [142] and taking siestas has been a cultural practise for centuries [144], these interventions have yet to be investigated with workers in real life settings and therefore empirical confirmation of these methods are still required. Additionally, these methods may not be implemented in industries with fixed timeframes and could interfere with the non-work related activities of the workers.

### Hydration and nutrition

Maintaining hydration was found to be modestly effective both at reducing physiological strain [93] as well as improving physical performance [67, 68, 85, 93]. This is likely because maintaining hydration does not actually provide any cooling (i.e. reducing core temperature when a worker is already overheating), but rather helps to limit physiological strain by replacing fluids lost to sweat. Further, as 2% dehydration is the classical threshold for decrements in physical performance [146], most of the studies analysed had participants dehydrated close to this level but not further [67, 70, 85, 91, 96, 98]. In contrast, protocols extending dehydration between 3 and 4% found reductions in cognitive performance between 23 and 25% [147, 148]. Importantly, typically morbidities and mortalities from heat stress are not due to the heat itself but the physiological responses to the heat, and cardiovascular issues, which are worsened by dehydration, in particular [2]. Further, a dramatic rise in chronic kidney disease associated with occupational heat strain related chronic dehydration has been well documented [8, 149, 150]. Accordingly, while hydration may not seem as important as other cooling strategies based on the present review, its role in long-term health is essential. Further, dehydration impairs cognition [98] and this may account for the increased incident rates of work site injuries during periods of hot weather [11]. As there is minimal additional cost, environmental impact, or extra effort required to provide workers with fluids (indoors) maintaining hydration should be prioritized and can be accomplished by working with the workers’ traditional diets [151]. For outdoor workers, efforts should be made to ensure adequate hydration via water bottles, water caches or backpacks containing water bladders [115].

As discussed in the qualitative review by Heffernan and colleagues [76], sodium consumption was found to help maintain hydration status and physical performance during physical exertion in the heat. When humans sweat, not only is water lost but so too are electrolytes. While excessive salt consumption is associated with negative health outcomes, namely elevated blood pressure [152], electrolytes are in fact critical both for blood pressure and hydration regulation within the body, as well as for the sweating process itself. Therefore, with prolonged sweating, if only water losses are replaced without additional electrolyte consumption, issues may arise with maintaining appropriate blood sodium levels and hydration status [153]. Indeed, case reports of life-threatening hyponatremia due to occupational heat strain with insufficient sodium replacement have been documented [154]. It is therefore critical that during work that elicits heavy sweating, lasting for several hours, workers should ingest sport drinks containing balanced electrolytes, as is presently recommended [143] . This information can likely be freely given to healthy workers but should be overseen by a physician for those workers with known blood pressure or other cardiovascular issues [155].

Making specific alterations to the diet in order to attempt to improve athletic performance has received much attention, however, how specific diets could potentially improve health and performance within the context of occupational heat strain has been largely unexplored. Increased carbohydrate ingestion is well known to improve athletic performance, however, one review which performed a meta-regression on carbohydrate supplementation during aerobic exercise found that the performance benefits of carbohydrate ingestion were reduced by 0.5% for every 10 °C increase in ambient temperature [107]. Protein ingestion has been less studied, however, one review on tyrosine supplementation [87] identified one study reporting an improvement in endurance physical performance in the heat while two studies observed no performance benefits. However, it is important to note that morphology and physiology differs considerably between athletes and everyday workers, and therefore, the effectiveness of these recommendations may differ. Further, high protein diets have been discouraged during hot weather, due to greater consequential urine output and water ingestion requirements [143]. Collectively, the present available data is not sufficiently strong to recommend specific alterations to the diet to help mitigate occupational heat strain, however, this is an interesting area of research for future research.

### Timing and location of the interventions

In terms of both mitigating physiological strain and improving performance, cooling before [66, 80, 89, 90, 92, 93], as opposed to during [72, 75, 80, 89, 90], physical activity in the heat was found to be more effective (Fig. 3). This finding may be due to the types of cooling interventions available before compared to during activity (e.g. whole-body cold water immersion was the most effective personal cooling intervention but cannot be employed during activity). This may also be because cooling interventions applied during exercise reduce the natural heat loss responses [156], whereas at rest, when cooling responses are not yet activated and cannot be reduced, cooling interventions allow for the reduction of core temperature, resulting in an internal heat sink [133, 135]. It is also important to note that in most of the original investigations contributing to the systematic reviews, most exertional protocols lasted between 1 and 2 h, and therefore activities of longer durations (such as an + 8 h work day), the internal heat sink effect may play a less significant role. Conversely, metabolic rates during occupational tasks are typically lower than those observed during athletic events [157], and therefore, the beneficial effects of precooling may be extended. Further, as the original studies informing these reviews were generally of an athletic context, clothing conditions differed considerably to real-world conditions (with less clothing generally being warn during athletics). However, it should be noted that precooling with ice slurries and cold water immersion has been found to be highly effective for short duration occupational tasks requiring highly insulating PPE (e.g. firefighters, those wearing hazmat suits, etc.) [158].

Additionally, in the one review looking at the effect of cooling location on physical performance [75], face cooling was found to be the most important cooling location, followed by the torso, and internal cooling. This effect was likely due to differences in human thermal comfort, as thermal sensation of the face [159] and back [160] have been demonstrated to have the greatest impact on thermal comfort in the heat. As with purely perceptual cooling (i.e. menthol), it is important to note that while selectively cooling specific areas may be beneficial for improving physical performance due to greater perceptions of comfort [161], it does not necessarily provide physical cooling and therefore creates the risk of over exertion in the heat.

### Present intervention evaluation compared to other evaluation hierarchies

For this review, we based our intervention-critique on previously established norms for evaluating the effectiveness of public health recommendations [26], whereas many occupational health and safety organisations use the Hierarchy of Controls, developed by NIOSH [162]. The Hierarchy of Controls categorises interventions (in descending order of desirability) as elimination, substitution, engineering controls, administration controls and PPE controls [162]; with the assumption that the interventions within each category have generally similar levels of effectiveness, cost, and feasibility. Following the Hierarchy of Controls method to evaluate cooling methods, which has been performed elsewhere [163], cooling interventions can typically be sorted as engineering (e.g. ventilators and radiation screens), administrative (e.g. work rest-schedules and rescheduling tasks) and PPE (e.g. cooling vests and ventilated clothing). In some areas, our public health evaluations agree well with this Hierarchy of Controls, as we found interventions that reduced the environmental heat stress (i.e. engineering control) to be most effective, on the contrary, we found clothing (i.e. PPE control) to be the second most effective method for cooling. Additionally, when considering cost, many of the most effective interventions become essentially unusable due to the exorbitant costs; an issue of particular concern in countries lacking occupational safety legislation and enforcement. Further, the Hierarchy of Controls model relies heavily on expert knowledge, conflicting with the present strength of evidence evaluation; however, this does provide the benefit of being more flexible (especially considering the large lack in occupation-specific knowledge identified by the present review) and specific to a given occupational environment. Both evaluation methods have strengths and weaknesses and indeed in the future, especially when more of the gaps in research are filled, the two methods could likely be used together to create better informed guidelines.

### Other issues raised by the review

The present review also identified several major gaps in the literature including a limited number of female participants, interactions between different cooling interventions, dietary interventions, characterization of the real-life/occupational physiological heat acclimatization effect, occupation-specific research, field-based research, and experimental protocols covering exposure/duration of relevance for a full working day. The gender discrepancy of the original study included in the umbrella review was very large, as 11,510 (91%) of the participants were male and only 1204 (9%) were female. This ratio is much worse than previous mass examinations of sports medicine/physical activity research that found approximately 61% of all participants were male and 39% were female [164]. This disproportionately large bias may be due to thermoregulatory studies often purposefully omitting female participants, due to the large fluctuations in core temperature that occur due to the menstrual cycle [165]. Although this finding further highlights the issue of lack of knowledge of the effectiveness of interventions in women, it does to some degree reflect the gender disparities that exist in labour intensive careers. For example, in the United States, females only represent 1% of stone masons, 2% of carpenters, 3–7% of construction workers, ~ 15% of metal workers, 16–22% of transportation workers, 20–25% of agricultural workers and ~ 30% of production workers [166]. Alternatively, in Bangladesh, females represent ~ 10% of plant and machine operators and assemblers; ~ 30% of crafts and related trades; and ~ 50% of skilled agriculture, forestry and fishery workers [167].

In terms of interventions lacking original research, some of the most pressing interventions include real-life/ occupational (physiological) heat acclimatization, interactions between different cooling interventions, and dietary interventions. As stated above, the heat acclimation/acclimatization literature typically focuses on athletes and therefore does not translate well to occupational settings. Some data suggests that heat acclimatization will be interfered with by spending time in air-conditioned spaces [123], whereas other data has demonstrated that 1 h of daily exercise in hot environmental conditions, while living in a northern hemispheric country during winter, is sufficient to initiate and maintain heat acclimation responses [168]. As such, to achieve an appropriate heat acclimatization response, there is likely a minimal threshold of internal and external thermal stress to cause adaptation responses, but that threshold is presently unknown. Similarly, recent research has indicated that cooling interventions may interfere with each other, and their effects, therefore, should not be considered as additive [169]. Accordingly, more research is needed to examine which combination of cooling interventions are most effective. Nutritional strategies are often mentioned in heat health guidelines [143], however, we were unable to find any studies investigating specific nutritional interventions for aiding with heat stress within an occupational context. Further, as the physiological states/demands of athletes and occupational workers differ significantly [170], nutritional needs of workers and athletes likely differ significantly and how well dietary recommendations from athletic literature is unknown.

As for the lack of occupation-specific, field-based research, as well as research protocols lasting longer than 2 h of duration, this likely stems from a tendency for thermal physiology research to focus on sport rather than occupational performance, likely due to the obvious concerns of high internal temperatures experienced during athletic activities. Indeed, only nine of the 36 reviews included in our analysis mentioned occupational heat strain, and of these reviews, the sole occupations of interest were military, firefighters, and emergency responders. In contrast, most research to date focusing on occupational heat strain has been largely based in epidemiology, climatology and public health, often focusing on the negative health consequences and predictions of labour losses, rather than developing solution for occupational heat strain [8, 9, 17, 124]. Indeed, a recent publication in *The Lancet* came to a similar conclusion regarding the lack of physiologic research in informing solutions for heat related deaths and illnesses [171] and has specifically made a call for greater work in this area. We hope this review may serve a similar purpose in highlighting the need for evidence-based recommendations for mitigating occupational heat strain.

## Conclusions

Complete cooling of the local environment is clearly the most effective way to reduce environmental heat stress, but presently, the reliance on fossil fuels and the high environmental impact of production, combined with very low feasibility in large production bays with high industrial heat production, or limited applicability in outdoor occupations, renders air-conditioning an unsuitable solution in most occupational environments. Fan use, alternatively, is highly effective, relatively low cost and feasible in more environments and providing shading outdoors during rest is likely beneficial. Following environmental cooling, clothing has the next greatest effect on health and performance. For highly stressful environments, cooling vests should be prioritized as they are effective, only moderately expensive and feasible in most environments. However, workers and employers are first encouraged to try and optimize existing clothing and/or PPE where possible/acceptable, by wearing light, brightly/lightly coloured or reflective, loose fitting and breathable clothing (when chemical or biological threats are not an issue), that covers the entire body outdoors to protect from solar radiation. Alternatively, when chemical or biological threats are not an issue, PPE should be worn that is sufficiently loose to ventilate and has incorporated higher air permeable patches to improve heat loss. Improving workers’ adapted physiological state was found to be very effective for improving work performance and mitigating physiological strain. While improving physical fitness of manual labour workers may be challenging, this finding demonstrates yet another reason to encourage being physically active and healthy. Further, heat acclimatization will likely occur naturally but sufficient time (1–2 weeks) is required before workers will benefit. For personal cooling strategies, ingestion of, and immersing in, cold water is most effective and occurs at modest costs and environmental impact. Although cold water immersion is less feasible, it can be accomplished by communal forearm cooling troughs during planned breaks. A generally under investigated, yet likely effective, inexpensive and feasible intervention, is to utilize pre-planned breaks in combination with the cooling interventions mentioned above, as well as to reorganize the work schedule to cooler times of the day. Further, maintaining hydration is important for maintaining cognitive and physical performance, mitigating physiological strain, and preventing long-term health consequences. Finally, areas most requiring future investigations include field and longer duration investigations, studies employing female participants, and currently understudied occupations (e.g. agriculture, construction and manufacturing).

## Supplementary information

**Additional file 1: Appendix 1.** search terms by category based on PICO table used for searching the databases. **Appendix 2.** Physiological effect size comparisons. **Appendix 3.** Physical performance effect size comparisons. **Appendix 4.** Cognitive performance effect size comparisons. **Appendix 5.** Perceptual response effect size comparisons.

## Data Availability

All data used in the manuscript are available from the articles cited and within the tables within the manuscript and appendices.
