# Supplementary material for: Sustainable solutions to mitigate occupational heat strain – an umbrella review of physiological effects and global health perspectives
Source: Environ Health. 2020 Sep 4;19:95. doi: 10.1186/s12940-020-00641-7 (PMC7487490; doi:10.1186/s12940-020-00641-7)
Supplement: Supplementary file 1 — Additional file 1: Appendix 1. search terms by category based on PICO table used for searching the databases. Appendix 2. Physiological effect size comparisons. Appendix 3. Physical performance effect size comparisons. Appendix 4. Cognitive performance effect size comparisons. Appendix 5. Perceptual response effect size comparisons. [file 12940_2020_641_MOESM1_ESM.docx]

Appendix 1. search terms by category based on PICO table used for searching the databases

| Stressors |  | Intervention |  | Outcome variables |  | Article type |
| --- | --- | --- | --- | --- | --- | --- |
| ((((((((((((heat stress) OR occupational) OR workplace) OR exercise) or work) OR occupation) OR labour) OR environment) OR ambient) OR aerobic) OR staff) OR job) OR hyperthermia | AND | ((((((((((((((((((((((((((((((((((((cooling intervention) OR cooling break) OR cooling) OR break) OR cold water) OR cold water immersion) OR cold water ingestion) OR ice slurry ingestion) OR skin wetting) OR skin misting) OR misting) OR spray) or menthol) OR air conditioning) OR dehumidification) OR fan) OR convection) OR HVAC) OR shading) OR hydration) OR nutrition) OR clothing) OR work-rest) OR work cycle) OR supplementation) OR pharmaceutical) OR drug) OR hat) OR compression garment) OR intermittent) OR electrolyte) OR caffeine) OR acetaminophen) OR nitrate) OR acclimation) OR acclimatisation) OR acclimatization | AND | (((((((((((core temperature) OR rectal temperature) OR skin temperature) OR sweat) OR thermal perception) OR perceived cooling) OR thermal comfort) OR exercise performance) OR cognition) OR cognitive performance) OR performance) OR productivity | AND | Review |

Appendix 2. Physiological effect size comparisons

| **Comparison** | **1st author  (year)** | **Studies in analysis** | **Physiological comparisons [ES (95%CI)]** | **Quality assessment** |
| --- | --- | --- | --- | --- |
| **Personal cooling** | | | | |
| Cold water immersion (pre) | Choo (2018) | 14 | Tc - Pre-cool: -1.24 (-2.00, -0.47)  Peak: -0.93 (-1.18, -0.67)  Change: +0.19 (-0.22, +0.6)  Tsk - Pre-cool: -4.48 (-5.98, -2.99)  Peak: -0.67 (-1.52, +0.18)  Change: +4.15 (+3.10, +5.21)  HR - Peak: -0.14 (-0.38, +0.11)  Change: +1.02 (+0.34, +1.69)  WBSR: -0.74 (-1.18, -0.30) | 8 |
| Ice ingestion (pre) | Choo (2018) | 11 | Tre - Pre-cool: -1.48 (-1.79, -1.18)  Peak: -0.10 (-0.48, +0.28)  Change: +1.02 (+0.59, +1.45)  Tsk - Pre-cool: -0.24 (-0.57, +0.10)  Peak: +0.10 (-0.22, +0.41)  Change: +0.34 (+0.02, +0.67)  HR - Peak: +0.08 (-0.19, +0.35)  Change: +0.26 (-0.01, +0.53)  WBSR: -0.12 (-0.42, +0.18) | 8 |
| Internal cooling | Best (2018) | 15 | Tre: -0.47 (-0.68, -0.26†) | 8 |
| Cold fluid ingestion | Burdon (2010) | 7 | Tre: -0.65 (-1.22, -0.07)# | 8 |
| **State of physiological adaptation** | | | | |
| Aerobic fitness | Alhadad (2019) | 22 | Tc: Pre: -0.65 (-0.85, -0.46) Change: +0.03 (-0.18, +0.24) Limit: +1.11 (+0.71, +1.51) | 6 |
| All HA | Tyler (2016) | 41 11 14 14 14 22 15 N/A 24 | Tre - Rest: -0.64 (-0.79, -0.50) Mean: -0.51 (-0.77, -0.26) Fixed point: -1.02 (-1.25, -0.78) Tsk - Rest: +0.07 (-0.16, +0.02) Mean: -0.85 (-1.22, -0.48) HR - Rest: -0.64 (-0.82, -0.46) Mean: -0.87 (-1.11, -0.64) Fixed point: -1.02 (-1.30, -0.74) WBSR: +0.61 (+0.42, +0.81) | 10 |
| STHA | Tyler (2016) | 6 | Tre: Rest: -0.61 (-0.85, -0.37) HR: Rest: -0.58 (-0.95, -0.21) WBSR: 0.24 (-0.04, +0.52) | 10 |
| MTHA | Tyler (2016) | 13 | Tre: Rest: -0.65 (-0.84, -0.47)  HR: Rest: -1.46 (-2.00, -0.91)  WBSR: 0.95 (+0.67, +1.24) | 10 |
| LTHA | Tyler (2016) | 2 | Tre: Rest: -0.93 (-1.82, -0.04)  HR: Rest: -1.46 (-2.00, -0.91)  WBSR: +0.95 (+0.06, +1.84) | 10 |
| Heat acclimation | Alhadad (2019) | 35 | Tc: Pre: -0.72 (-0.86, -0.58) Change: -0.35 (-0.19, -0.50) Limit: -0.28 (-0.52, -0.04) | 6 |
| Heat acclimation | Rahimi (2019) | 7 5 4 11 5 | HR: SS: -0.68 (-1.87, +0.51) HR: TT: +0.76 (+0.23, +1.29) HR: MAX: -6.57 (-12.79, -0.35) Tc: -0.03 (-0.13, +0.06)  Tsk: -0.31 (-0.68, +0.06) | 8 |
| **Clothing** | | | | |
| Compression garments | Born (2013) | 6 | T_c_: +1.38 (+0.63, +2.13) | 8 |
| **Hydration** | | | | |
| Fluid intake | Alhadad (2019) | 24 | Tc: Pre: -0.11 (-0.31, +0.08) Change: -0.70 (-0.94, -0.46) Limit: -0.50 (-0.74, -0.27) | 6 |
| **Timing of intervention** | | | | |
| Pre | Alhadad (2019) | 42 | Tc: Pre: -1.01 (-1.17, -0.85) Change: +0.46 (+0.28, +0.63) Limit: -0.29 (-0.44, +0.14) | 6 |
| Pre | Best (2018) | 4 | Tre: -0.42 (-0.93, 0.10) | 8 |
| Per | Ruddock (2017) | 10 10 10 7 6 | Tre - mean: -0.08 (-0.37, +0.22)  end: -0.21 (-0.47, +0.04)  Tsk - mean: -0.28 (-0.56, 0.00)  HR - mean: -0.03 (-0.37, +0.32)  WBSR: -0.13 (-0.50, 0.23) | 9 |
| Per | Best (2018) | 8 | Tre: -0.37 (-0.65, -0.10†) | 8 |
| Mixed | Best (2018) | 8 | Tre: -0.59 (-0.90, +0.28) | 8 |

ES: effect size; 95%CI: 95 percentile confidence intervals; Tc: core temperature; Tre: rectal temperature; Tsk: skin temperature; HR: heart rate; WBSR: whole-body sweat rate. † denotes 90th percentile confidence intervals. # denotes ES calculated from original study data. + denotes increase (worsening) in physiological response. – denotes reduction (improvement) physiological response. Quality assessment scores from the AMSTAR checklist results in Table 2.

Appendix 3. Physical performance effect size comparisons

| **Comparison** | **1st author  (year)** | **Studies in analysis** | **Physical performance**  **comparisons [ES (95%CI)]** | **Quality assessment** |
| --- | --- | --- | --- | --- |
| **Environmental manipulation** | | | | |
| Air ventilation | Douzi (2019) | 5 | +0.91 (+0.26, +1.52) | 8 |
| **Personal cooling** | | | | |
| Cold water immersion (pre) | Choo (2018) | 14  9 5 | All: +0.53 (+0.28, +0.77)  Self-paced: +0.35 (+0.06, +0.64) TTE: +1.06 (+0.37, +1.76) | 8 |
| Skin wetting | Best (2018) | 1 | +0.20 (-0.53, +0.94†) | 8 |
| Cold water immersion (pre) | Bongers (2015) | 5 | +0.49 (+0.09, +0.90) | 7 |
| Cooling packs (pre) | Bongers (2015) | 2 | +0.40 (+0.10, +0.71) | 7 |
| Cooling packs (per) | Bongers (2015) | 7 | +0.34 (+0.09, +0.58) | 7 |
| Cooling packs | Chan (2015) | 2 | +0.13 (-0.60, +0.86) | 9 |
| Face and head cooling | Chan (2015) | 6 | +0.36 (-0.06, +0.78) | 9 |
| Cold water immersion (pre) | Jones (2012) | 3 2 1 6 | Time: +1.63 (+0.45, +2.82) Distance: +0.52 (-0.52, +1.56) MPO: +0.74 (-0.28, +1.77) Pooled: +1.11 (0.00, +2.23) | 11 |
| Neck collar (per) | Douzi (2019) | 6 | +0.48 (+0.22, +0.70) | 8 |
| Cold water immersion | Douzi (2019) | 1 | +0.26 (-0.17, +0.65) | 8 |
| Hand cooling devices | Douzi (2019) | 3 | +0.52 (-0.17, +1.22) | 8 |
| Water spray | Douzi (2019) | 1 | +0.17 (-0.43, +0.78) | 8 |
| Ice ingestion (pre) | Choo (2018) | 11  8 3 | All: +0.2 (-0.07, +0.46) Self-paced: +0.13 (-0.18, +0.45) TTE: +0.37 (-0.15, +0.88) | 8 |
| Internal cooling | Best (2018) | 15 15 | +0.39 (+0.18, +0.60†) PO: +0.22 (-0.22, +0.66†) | 8 |
| Cold water ingestion (pre) | Bongers (2015) | 6 | +0.40 (+0.17, +0.62) | 7 |
| Cold water ingestion (per) | Bongers (2015) | 1 | +1.75 (+0.38, +3.12) | 7 |
| Ice slurry ingestion (pre) | Jones (2012) | 4 2 6 | Time: +0.66 (-0.32, +1.63) MPO: +0.38 (-0.58, +1.33) Pooled: +0.57 (-0.40, +1.53) | 11 |
| Cold drinks (pre) | Wegman (2012) | 18 | +1.61 (+0.39, +2.82) | 7 |
| Cold drinks | Burdon (2010) | 4 | +1.21 (-0.09, +2.51)# | 8 |
| Menthol | Douzi (2019) | 4 | +0.09 (-0.17, +0.52) | 8 |
| Internal menthol application | Jeffries (2019) | 6 | +0.40 (+0.04, +0.76) | 8 |
| External menthol application | Jeffries (2019) | 7 | +0.29 (-0.34, +0.91) | 8 |
| Pooled menthol application | Jeffries (2019) | 13 | +0.33 (0.00, +0.65) | 8 |
| **Hydration** | | | | |
| Fluid intake | Alhadad (2019) | 9 | -0.16 (-0.53, +0.22) | 6 |
| Fluid intake cycling performance | Holland (2017) | 15 | +0.25 (-0.13, +0.64) | 10 |
| Fluid intake cycling performance (<1 h) | Holland (2017) | 6 | -0.34 (-0.65, -0.04)# | 10 |
| Fluid intake cycling performance (>1 h, <2 h) | Holland (2017) | 3 | +0.98 (+0.02, +1.93)# | 10 |
| Fluid intake cycling performance (>2 h) | Holland (2017) | 6 | +0.48 (-0.01, +0.98)# | 10 |
| Fluid intake during continuous exercise following dehydration | McCartney (2017) | 18 | +0.46 (+0.32, +0.61) | 10 |
| Fluid intake during continuous exercise following dehydration in over 30°C | McCartney (2017) | 11 | +1.09 (+0.50, +1.69) | 10 |
| Time trial while dehydrated | Goulet (2011) | 13 | -0.17 (-0.42, +0.07)# | 8 |
| **State of physiological adaptation** | | | | |
| Aerobic fitness | Alhadad (2019) | 5 | +1.01 (+0.61, +1.40) | 6 |
| Heat acclimation | Alhadad (2019) | 7 | +0.19 (-0.16, +0.54) | 6 |
| Heat acclimation | Rahimi (2019) | 6 | TT: -0.68 (-1.89, +0.54) | 8 |
| Heat acclimation | Rahimi (2019) | 4 | VO2max: -2.51 (-6.37, +1.36) | 8 |
| All heat acclimation | Tyler (2016) | 26 26 | Performance: +0.65 (+0.46, +0.83) Capacity: +0.84 (+0.63, +1.04) | 10 |
| Short term heat acclimation | Tyler (2016) | 15 | +0.52 (+0.26, +0.78) | 10 |
| Medium term heat acclimation | Tyler (2016) | 25 | +0.75 (+0.57, +0.92) | 10 |
| Long term heat acclimation | Tyler (2016) | 4 | +0.93 (+0.46, +1.39) | 10 |
| **Clothing** | | | | |
| Cooling vest (pre) | Bongers (2015) | 6 | +0.19 (+0.10, +0.28) | 7 |
| Cooling vest (per) | Bongers (2015) | 1 | +4.64 (+0.96, +8.32) | 7 |
| Phase change cooling garments | Chan (2015) | 7 | +1.20 (+0.59, +1.80) | 9 |
| Cooling garments (pre) | Jones (2012) | 3 1 4 | Time: +0.28 (-0.54, +1.09) MPO: +0.16 (-0.97, +1.30) Pooled: +0.22 (-0.75, +1.20) | 11 |
| Air-cooled garment | Chan (2015) | 4 | +1.12 (0.49, 1.75) | 9 |
| Cold air-cooled garments | Chan (2015) | 4 | +2.32 (1.25, 3.39) | 9 |
| Liquid cooled garments | Chan (2015) | 4 | +1.86 (1.16, 2.56) | 9 |
| Hybrid cooling garment | Chan (2015) | 2 | +1.61 (-1.58, 4.81) | 9 |
| Ventilated vests | Douzi (2019) | 3 | +1.48 (-0.30, +3.26) | 8 |
| Ice vests | Douzi (2019) | 6 | +1.17 (+0.35, +2.00) | 8 |
| Hybrid vests | Douzi (2019) | 1 | +0.17 (-0.17, +0.48) | 8 |
| Cooling garments | Douzi (2019) | 1 | +1.83 (+0.17, +3.48) | 8 |
| **Mixed cooling methods** | | | | |
| Mixed methods | Best (2018) | 4 | +0.07 (-0.29, +0.44†) | 8 |
| Mixed methods (pre) | Jones (2012) | 2 4 6 | Time: +0.62 (-0.42, +1.65) MPO: +0.39 (-0.61, +1.39) Pooled: +0.49 (-0.53, +1.50) | 11 |
| Mixed method (pre) | Bongers (2015) | 8 | +0.72 (+0.49, +0.96) | 7 |
| **Timing of applied cooling interventions** | | | | |
| Net cooling (per) | Douzi (2019) | 38 | +0.60 (+0.43, +0.77) | 8 |
| Pre | Alhadad (2019) | 24 | -0.20 (-0.56, +0.17) | 6 |
| Pre | Bongers (2015) | 27 | +0.44 (+0.31, +0.56) | 7 |
| Pre | Jones (2012) | 22 | +0.63 (-0.38, +1.63) | 11 |
| Pre | Wegman (2012) | 19 | +0.67 (-0.31, +1.64) | 7 |
| Pre | Tyler (2015) | 60 | +0.77 (0.65, +0.89) | 10 |
| Pre (intermittent) | Tyler (2015) | 26 | +0.47 (+0.40, +0.53) | 10 |
| Pre (prolonged) | Tyler (2015) | 16 | +2.04 (+1.53, +2.36) | 10 |
| Pre (capacity) | Tyler (2015) | 6 | +1.13 (+1.00, +1.25) | 10 |
| Pre | Best (2018) | 4 | +0.13 (-0.27, +0.52†) | 8 |
| Per | Bongers (2015) | 9 | +0.40 (+0.15, +0.66) | 7 |
| Per | Ruddock (2017) | 11 8 3 | Pooled: +0.48 (+0.18, +0.78) Preload: +0.47 (+0.12, +0.83)  No-preload: +0.50 (+0.04, +1.04) | 9 |
| Per | Tyler (2015) | 5 | +0.72 (+0.58, +0.94) | 7 |
| Per (compensable) | Tyler (2015) | 4 | +0.46 (+0.40, +0.50) | 7 |
| Per (uncompensable) | Tyler (2015) | 1 | +2.26 | 7 |
| Per | Best (2018) | 8 | +0.21 (-0.05, +0.48†) | 8 |
| Mixed | Best (2018) | 8 | +0.44 (+0.18, +0.69†) | 8 |
| **Cooling location** | | | | |
| Mouth rinse | Douzi (2019) | 2 | +0.04 (-0.43, +0.52) | 8 |
| Internal cooling | Douzi (2019) | 7 | +0.52 (+0.26, +0.78) | 8 |
| Neck cooling | Douzi (2019) | 6 | +0.43 (+0.22, +0.70) | 8 |
| Hand cooling | Douzi (2019) | 4 | +0.35 (-0.09, +0.78) | 8 |
| Face cooling | Douzi (2019) | 4 | +1.22 (0.52, +1.91) | 8 |
| Torso cooling | Douzi (2019) | 10 | +0.96 (+0.43, +1.48) | 8 |
| Whole body cooling | Douzi (2019) | 5 | +0.43 (0.00, +0.87) | 8 |

ES: effect size; 95%CI: 95 percentile confidence intervals; TTE: time to exhaustion; PO: power output; MPO: mean power output; # ES calculated from original study data. † denotes 90th percentile confidence intervals. + denotes improved performance. – denotes reduced performance. Quality assessment scores from the AMSTAR checklist results in Table 1.

Appendix 4. Cognitive performance effect size comparisons

| **Comparison** | **1st author  (year)** | **Studies in analysis** | **Cognitive comparisons [ES (95%CI)]** | **Quality assessment** |
| --- | --- | --- | --- | --- |
| **Hydration** | | | | |
| Overall effect of dehydration on cognitive performance | Wittbrodt (2018) | 33 | -0.21 (-0.31, -0.11) | 5 |
| Attention tasks | Wittbrodt (2018) | 10 | -0.54 (-0.69, -0.39) | 5 |
| Motor coordination | Wittbrodt (2018) | 5 | -0.40 (-0.63, -0.17) | 5 |
| Reaction time | Wittbrodt (2018) | 16 | -0.10 (-0.23, +0.03) | 5 |
| Overall cognitive function | Goodman (2019) | 10 | -2.61 (-0.69, +0.17) | 9 |
| Overall complex attention | Goodman (2019) | 6 | -0.11 (-0.39, +0.19) | 9 |
| Overall executive function | Goodman (2019) | 6 | -0.27 (-1.30, +0.77) | 9 |
| Overall learning and memory | Goodman (2019) | 8 | -0.27 (-0.79, +0.25) | 9 |

ES: effect size; 95%CI: 95 percentile confidence intervals; – denotes reduction (worsening) of cognitive performance with dehydration. Quality assessment scores from the AMSTAR checklist results in Table 2.

Appendix 5. Perceptual response effect size comparisons

| **Comparison** | **1st author  (year)** | **Studies in analysis** | **Perceptual comparisons [ES (95%CI)]** | **Quality assessment** |
| --- | --- | --- | --- | --- |
| **Personal cooling** | | | | |
| Cold water immersion (pre) | Choo (2018) | 14 | RPE: -0.14 (-0.39, +0.12)  TS: -0.50 (-0.80, -0.19) | 8 |
| Water dousing (pre) | Best (2018) | 1 | RPE: -0.50 (-1.03, +0.04†)  TMC: -1.35 (-2.18, -0.51†) | 8 |
| Ice ingestion (pre) | Choo (2018) | 11 | RPE: -0.01 (-0.33, +0.31)  TS: -0.20 (-0.49, +0.10) | 8 |
| Internal cooling | Best (2018) | 15 | RPE: -0.41 (0.61, -0.20†) | 8 |
| Mixed methods | Best (2018) | 4 | RPE: -0.13 (-0.53, +0.27†)  TS: -0.36 (-1.25, +0.53†) | 8 |
| **Heat acclimation** | | | | |
| All HA | Tyler (2016) | 3 4 3 4 | RPE: -0.63 (-1.09, -0.17) TS - Rest: +0.04 (-0.40, +0.48) Mean: -0.68 (-1.17, -0.18) Fixed point: -0.38 (-0.78, -0.03) | 10 |
| **Menthol application** | | | | |
| Internal menthol application | Jeffries (2019) | 5 | TS: -0.30 (-0.50, -0.10) | 8 |
| External menthol application | Jeffries (2019) | 6 | TS: -0.71 (-0.88, -0.54) | 8 |
| Pooled | Jeffries (2019) | 11 | TS: -0.54 (-0.67, -0.42) | 8 |
| **Timing of intervention** | | | | |
| Pre | Best (2018) | 4 | RPE: +0.17 (-0.18, 0.52) | 8 |
| Per | Ruddock (2017) | 8 6 | RPE: -0.49 (-0.81, -0.17)  TS: -0.67 (-1.06, -0.29) | 9 |
| Per | Best (2018) | 8 | RPE: -0.39 (-0.70, -0.08†)  TS: -0.60 (-1.51, 0.31†)  TMC: 1.29 (-0.82, +1.76†) | 8 |
| Mixed | Best (2018) | 8 | RPE: -0.48 (-0.75, -0.22) | 8 |

ES: effect size; 95%CI: 95 percentile confidence intervals; RPE: rating of perceived exertion; TS: thermal sensation; TMC: thermal comfort. † denotes 90th percentile confidence intervals. + denotes increase (worsening) in perceptual response. – denotes reduction (improvement) perceptual response. Quality assessment scores from the AMSTAR checklist results in Table 2.
